# Enzymology of the metazoan tRNA ligase complex: a lifetime in cycles

**DOI:** 10.1007/s00018-026-06133-0

**Published:** 2026-04-11

**Authors:** Igor Asanović, Javier Martinez

**Affiliations:** Max Perutz Labs, Medical University of Vienna, Vienna BioCenter (VBC), Dr. Bohr-Gasse 9/2, Vienna, 1030 Austria

**Keywords:** tRNA-LC, RTCB, DDX1, PYROXD1, Archease, Ashwin, tRNA

## Abstract

This review highlights the emerging biochemistry and biology of the metazoan tRNA-ligase complex (tRNA-LC). We begin with an overview of the cleavage-ligation pathways dependent on the tRNA-LC, epitomised by the essential process of pre-tRNA splicing, but also critical for the unfolded protein response and the struggle against transposons. The catalytic core of the tRNA-LC is the non-conventional RNA ligase RTCB, found in all domains of life. We start from the universal principles of its catalytic cycle, entailing GTP-dependent ligation of RNA molecules with 2’,3’-cyclic phosphate and 5’-hydroxyl ends. We then focus on the new findings that govern trafficking, protection, regulation and degradation of the tRNA-LC. These new modalities arise from an expanded set of subunits that to a large extent specifically associate with RTCB in *Eukarya*. We present how co-purification and co-evolutionary analyses converged to guide sequential discoveries of these proteins and illuminated their biochemical roles. We detail how the choice of paralogue of the auxiliary subunit FAM98 and the recruitment of Ashwin determine RTCB localisation, discriminating between its cytoplasmic and nuclear roles. We then pay particular attention to discoveries emerging from the latest structural works. These include the Archease-mediated mechanism of metazoan RTCB guanylylation, and PYROXD1-mediated protection of the tRNA-LC in the “resting state” from oxidative inactivation. We illustrate how metal ions play critical roles in both of these processes, alongside their direct roles in catalysing RNA ligation and, potentially, in the degradation of the tRNA-LC through a novel and still mysterious mechanism. We place particular emphasis on the subunits with contested functions, and on the interplay between their role in the tRNA-LC and their other cellular tasks. Along the way, we highlight the links between the subunits of the tRNA-LC, in particular their pathogenic variants or their mis-localisation, and human diseases including neurodegeneration and myopathies. The increasing understanding of the tRNA-LC, including its expanded set of subunits, illustrates how branching out from a single enzyme uncovered new biology ranging from the previously unknown congenital myopathies to novel proteasome targeting pathways.

## RNA 2’,3’ cyclic phosphate ligase RTCB: an ancient enzyme with modern challenges

Eukaryal RNA splicing, mediated by the megadalton spliceosome machinery, is a reaction that increases the functional output of genomes by allowing single genes to encode for multiple protein isoforms [1, 2]. This is achieved through selective removal of parts of RNA molecules known as “introns” with the concomitant joining of the remaining “exonic” sequences in varying combinations [the nomenclature introduced in [3]]. The discovery of RNA splicing revolutionised biology by breaking the paradigm postulating that every polypeptide is encoded by a distinct gene. Spliceosomes have been extensively studied on biochemical and structural levels despite their complexity, and their functions are textbook knowledge [4–7].

Yet, they are neither the only, the oldest, nor the most widespread enzymatic system capable of cleaving and re-ligating RNA molecules. All three domains of life employ spliceosome-independent RNA cleavage and ligation reactions for various purposes [8–11]. In contrast to the spliceosome-mediated splicing, spliceosome-independent cleavage and ligation are two distinct reactions that give rise to (potentially accumulating) cleaved intermediates. Furthermore, while RNA moieties provide important catalytic contribution to the spliceosomes [12, 13], the alternative cleavage-ligation reactions are often exclusively facilitated by protein catalysts. They are necessary for fundamental processes such as biogenesis of transfer RNAs (tRNAs) [reviewed in [14–16], as well as for the conditional reactions required for responses to or recovery from different stress conditions [17, 18].

The opening section of this review will briefly describe these cleavage-ligation pathways and introduce an RNA ligation mechanism operating across all domains of life.

**Table:**
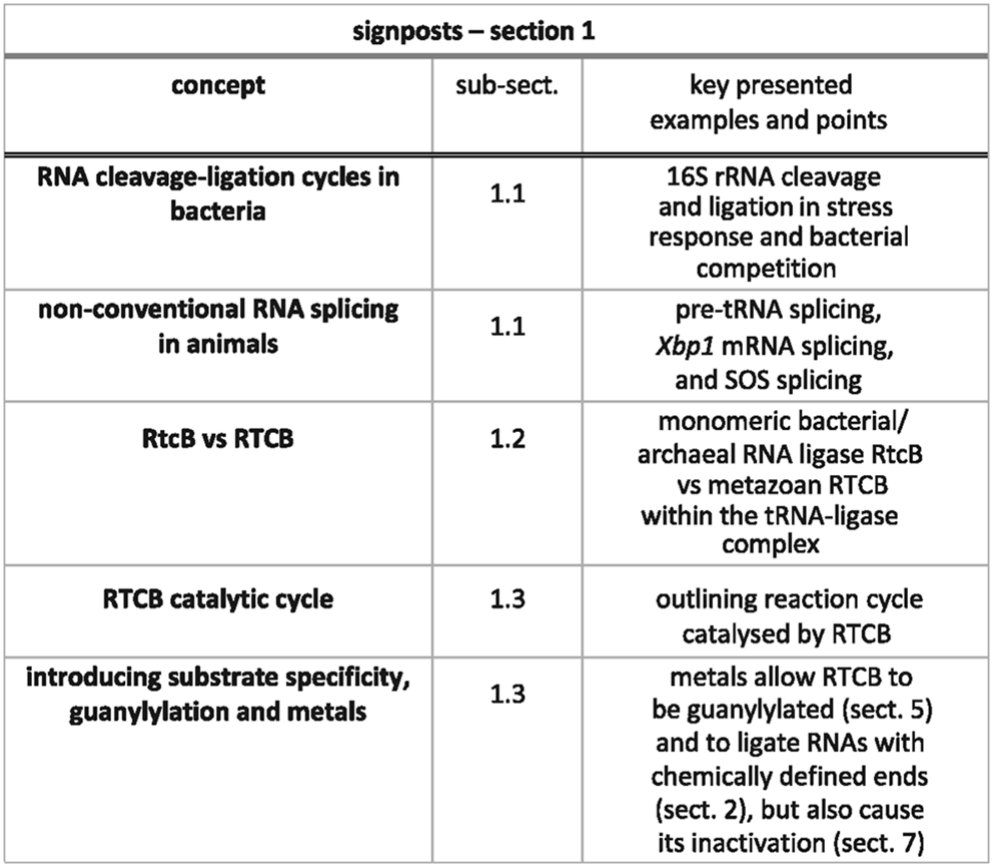

### RNA cleavage-ligation reactions from bacteria to animals

**Fig. 1:**
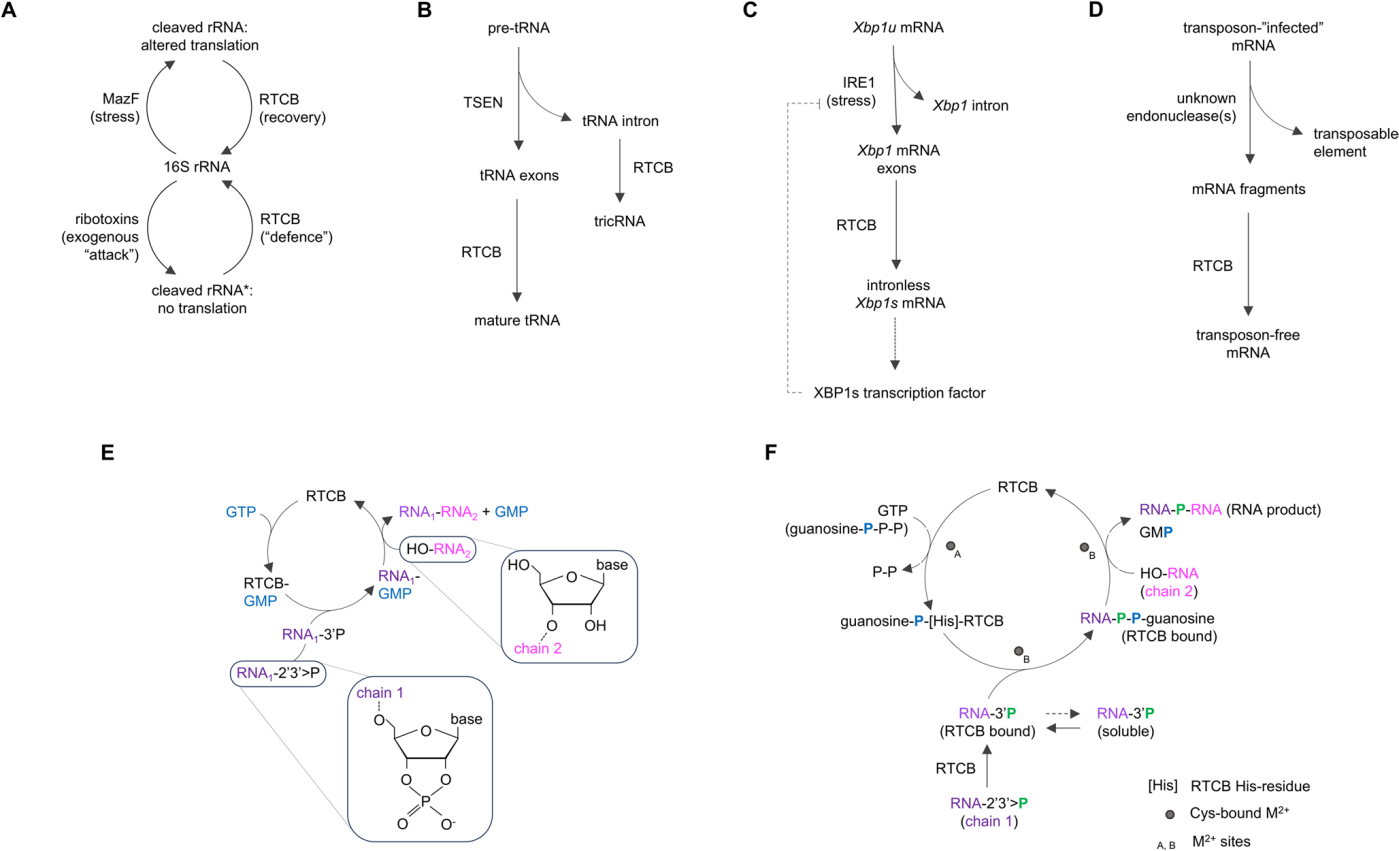
RtcB/RTCB-mediated non-canonical RNA splicing reactions and their conserved catalytic cycle. (**A**) Cleavage-ligation cycle in *E. coli* 16S rRNA during oxidative stress and recovery (top) and in the context of bacterial cell-cell competition (bottom), whereby one bacterial cell secretes ribotoxins that cleave 16S rRNA in its neighbours. * The cleaved products in (top) and (bottom) differ, as MazF cleaves upstream of anti-Shine-Dalgarno sequence, while the cut sites of the ribotoxins used in bacterial competition reside in the decoding centre. (**B**) Metazoan pre-tRNA splicing pathway. Intron excision from intron-containing pre-tRNAs is critical for their function, as only mature, spliced tRNAs are functional in translation. While the functions of stable, intronic tricRNAs remain largely elusive, they are sometimes used as activity readouts for RTCB in cells. (**C**) Metazoan non-canonical splicing of *Xbp1* mRNA. This pathway is triggered by stress at the ER and results in stress alleviation through a negative feedback loop. (**D**) Metazoan SOS splicing pathway. SOS splicing is a mechanism of defence against transposons, whereby the transposable element is excised (and presumably degraded) by a still elusive endonuclease(s), and the remaining mRNA fragments are subsequently ligated by RTCB to form a healed mRNA. (**E**) Simplified RTCB catalytic cycle, presenting the main steps and reaction participants; chemically specific RNA termini that define the canonical RTCB substrates are depicted with structural formulae (see panel F for more detailed dissection, including substrate selection, phosphate destinies and the roles of metal ions). (**F**) (left) The catalytic cycle is initiated by guanylylation of an invariant histidine residue, followed by (middle) the nucleophilic attack of the 3’P group attached to the end of an RNA strand. (bottom) 3’P is canonically generated via RTCB-mediated hydrolysis of 2’,3’>P, although the mammalian RTCB can also acquire it from solution in vitro. Finally, (right) OH group from the 5’ end of the second RNA strand attacks the RNA-P-P-guanosine intermediate to generate the ligated product and liberate GMP. Green and blue P denote phosphoryl groups originating from the RNA substrate and GTP, respectively. ** While the metals at specific sites were proposed to play critical roles in the denoted reactions [20], more recent findings imply that some steps likely rely on more than one ion. For example, while the metal in site A is critical to increase nucleophilicity of the GTP α-P, other metal ions assist in substrate positioning and leaving group stabilisation (see Fig. 5)

In *Bacteria*, a prominent example of a cleavage-ligation cycle is triggered in response to non-homeostatic events such as DNA damage. To this end, *Escherichia coli* utilises the endonuclease MazF to cleave the anti-Shine-Dalgarno (a-SD) sequence of 16S ribosomal RNA (rRNA) (Fig. 1A, top). Such “stress-ribosome” is retuned towards translation of specific stress-response transcripts, which are also subjects to MazF-mediated cleavage, and therefore lack SD sequences [19]. Upon stress recovery however, the rRNA is re-ligated, restoring the homeostatic ribosomal function [18].

 16S rRNA is also substrate of another cleavage-ligation cycle, occurring in the context of bacterial competition (Fig. 1A, bottom). Here, bacterial cells secrete toxic endonucleases that target the ribosomal decoding centre in their neighbours, rendering ribosomes non-functional in protein translation; the targeted cells counter such ribotoxins by repairing 16S rRNA through an RNA ligation reaction [21]. As both of the described reactions that target 16S rRNA in bacteria do not entail intronic excision, they are exclusively cleavage-ligation, or breakage-repair cycles rather than splicing events.

In contrast, a varying fraction of *archaeal* and *eukaryal* tRNA molecules are encoded with an intronic sequence that needs to be excised for the tRNA to function as decoder of messenger RNAs (mRNAs) in protein synthesis [15, 22] (Fig. 1B). The intron cleavage is catalysed by the tRNA splicing endonuclease (TSEN) complex [11, 23–25] [reviewed in [26, 27]]. As all transcribed Tyr^GTA^, Ile^TAT^ and Leu^CAA^ tRNAs in humans are synthesised with an intron, this reaction is required for the comprehensive decoding of the genetic code and is therefore essential for human life [28, 29].

Reactions critical for pre-tRNA splicing create new regulatory opportunities in tRNA biology [30–32] (see Sect. 7) and generate distinct tRNA fragments with impact on cell viability [33, 34] (see Sect. 2). The spliced intron may undergo circularisation, resulting in a stable product coined tRNA intronic circular RNA (tricRNA) with as of yet unknown roles [35] (Fig. 1B, middle). In yeast, non-circularised tRNA introns (“free introns of tRNAs”, fitRNAs) act in a manner comparable to microRNAs, mediating mRNA silencing via complementarity-based degradation [36]. Finally, other tRNA modifications rely on either retention or removal of the pre-tRNA introns, both in yeast [37, 38] and in animals [39]. An example with striking physiological impact is the intron-dependent ^m1^A58 modification of Tyr^GUA^ tRNA, which occurs at a position distal to the intron itself and prevents undesired ribosome collision and integrated stress response. This mechanism likely operates across *Eukarya*, given the near-universal presence of the intron in Tyr^GUA^ tRNA [40]. The full evolutionary benefits of pre-tRNA splicing [reviewed in [41], are yet to be fully explored.

A reaction mechanistically similar to pre-tRNA splicing occurs with a unique mRNA, *Xbp1*. When the endoplasmic reticulum (ER) is faced with stress, for example due to excessive burden for the synthesis of extracellular proteins such as antibodies, *Xbp1* mRNA is cleaved by the ER-transmembrane endonuclease IRE1 (Fig. 1C). This results in removal of an intron, and the remaining exons are subsequently ligated [42, 43] [reviewed in [44]]. Only the spliced mRNA form encodes for the transcription factor XBP1s, critical for the expression of proteins involved in countering ER stress and maintaining the ER homeostasis (Fig. 1C). Interestingly, the non-ligated 3’ exon of *Xbp1* was also shown to be functional, playing a key role in controlling axonal regeneration in a worm model [45]. Spliceosome-independent *Xbp1u* mRNA splicing is sometimes referred to as “non-canonical“ or “unconventional” RNA splicing [46–48] and can be contextualised among diverse purposeful break-repair pathways [16].

The most recently described “unconventional” RNA splicing reaction occurs in the context of transposon biology (Fig. 1D). In “SOS splicing”, a defence mechanism against transposons, transposable elements are first excised from the“infected” mRNAs by unknown endonuclease(s), followed by healing of the remaining pieces to create functional mRNA transcripts [49].

**Table:** 

Pro-transposon RNA ligation	
In contrast to “anti-transposon” SOS splicing, a non-conventinal RNA ligation reaction can also play a “pro-transposon” role, when hijacked by LINE-1 elements to assist in their retrotransposition. The substrates of this reaction are U6 snRNA and LINE-1 RNA, which form chimeric RNAs during retrotransposition events [116]. This reaction thus entails an atypical merger of two RNA entities of distinct origins, as opposed to the cleavage-ligation cycles (Fig. 1A-D) that are primary referred to in this review.	

### RtcB/RTCB: RNA healing enzyme(s)

The ligation step in all the above described examples occurs through a shared reaction mechanism. In *Bacteria* and many *Archaea*, it is catalysed by an individual protein, named RtcB [51]. *Eukarya* inherited this one-subunit enzyme, here designated as RTCB and historically also called HSPC117 [52]. The complexity of compartmentalised eukaryal cells however required a better regulated enzymatic system capable of acting across membrane-segregated subcellular compartments (see Sect. 4). Furthermore, oxygenic environment in which most modern *Eukarya* thrive is harmful for the highly vulnerable RTCB active site. Relying on RTCB for an essential cellular process such as tRNA biogenesis thus became a major liability (see Sect. 7). Consequently, genomes of many eukaryal phyla, including land plants and non-parasitic fungi, have abandoned RTCB to history. These organisms fully replaced RTCB with the phylogenetically and structurally unrelated RNA ligase Trl1 [8, 53–55], which is mechanistically rather analogous to the one employed by the bacteriophage T4 during host defence [56, 57]. The others, including all animals, have however retained RTCB.

Yet, this came with an evolutionary cost: instead than being encoded as a single self-sufficient medium-sized polypeptide, RTCB in animals relies on a set of at least eight tightly co-evolving proteins [31, 58], alongside the assistance from numerous general cellular factors. While metazoan RTCB retained the substrate specificity and the basic chemical steps of RNA ligation of the archaeal ancestor, these additional proteins make its enzymology more complex, and many of its aspects are only now being revealed or fundamentally re-addressed (see Sects. 3 and 6). Structural and mechanistic aspects of RtcB/RTCB biology, and the insights into pre-tRNA splicing across species have been covered in recent reviews [15, 16, 22, 59]. However, understanding of the metazoan RNA ligation has been rapidly expanding, revealing unique twists in its evolution and regulation, as well as contributions and pathological roles of the “accessory” proteins. We will therefore provide only a short overview of the general biochemistry of RTCB (sub-Sect. 1.3). Thereafter (Sects. 2–7), this review will primarily dissect the complexities of the metazoan tRNA-LC, which turn the unconventional RNA ligation in animals into a vibrant, diverse and biomedically relevant field.

We will first outline general principles of RTCB/RtcB-mediated RNA ligation reactions. These include recognition of the substrates and requirement for metal ions and GTP, together with the corresponding invariably conserved binding residues. For simplicity, we will refer to (metazoan) RTCB as a model and emphasise divergences with (bacterial or archaeal) RtcB where they occur. Where applicable, we will provide both the metazoan-specific, as well as the bacterial or archaeal references to illustrate the universality of RTCB/RtcB features.

### Universal principles of the catalytic cycle of RTCB

RTCB does not randomly ligate any RNA ends it encounters in the cell; rather, it primarily participates in dedicated cleavage-ligation pathways (see sub-Sect. 1.1). To achieve this, RTCB requires a narrow substrate specificity. Nucleotide sequence or secondary structures have so far not been observed as critical intrinsic determinants of RTCB-mediated reactions; in contrast, RTCB recognises chemically specific RNA termini, typically generated through the preceding cleavage reactions [52, 60, 61] (see Fig. 1A-D). In other words, the chemistry of RNA termini suitable for RTCB-mediated ligation needs to match those created by endonucleases such as TSEN and IRE1. These termini are 2’,3’ cyclic phosphate (here: 2’,3’> P) at the 3’ end, and 5’OH at the 5’ end of the cleaved RNA strands, depicted within the simplified RTCB reaction cycle in Fig. 1E (for more detail, see below) [24, 62]. The unique chemistries of RNA termini critical for all RTCB-mediated ligation reactions thus ensure the appropriate substrate recognition.

Ligation of RNA strands is typically an endergonic process. To facilitate its completion, RTCB couples the reaction with exergonic hydrolysis of GTP [58, 61, 63]. To this end, catalytic cycle is initiated by the nucleophilic attack of an invariant histidine residue (H428 in human RTCB) on the α-phosphoryl group of GTP with concomitant release of inorganic pyrophosphate and creation of a covalent, activated protein-GMP intermediate (Fig. 1E/F, top left; see section 5) [64]. The free energy is subsequently carried over to the next guanylylated intermediate, this time the RNA strand attached to a guanosine group via a pyrophosphate bridge, and ultimately released through the attack of the 5’OH group from the second RNA strand, resulting in the ligation of two RNA ends, liberation of GMP and recycling of RTCB for the next reaction cycle (Fig. 1E/F, top right). To enable the second reaction, RTCB first needs to hydrolyse 2’,3’>P via its cyclic phosphodiesterase activity to generate the 3’ phosphate (3’P) required for the attack onto the guanylylated protein [65] [reviewed in [59]].

3’P-ended RNAs can be directly supplied instead of 2’,3’> P as RTCB substrates in vitro [52, 61] (Fig. 1E/F, bottom). This implies that 3’P reaction intermediate could be permitted to dissociate and re-associate with the enzyme throughout the catalytic cycle, or be generated through other enzymatic activities (see Sect. 2). However, it is not known if such utilisation of dissociated or exogenously generated 3’P as substrate is permissible in cells, where RTCB may display a stronger preference for 2’,3’> P-ended RNAs.

**Table:** 

Refusing 3’P substate?	
Not all RtcB enzymes may be able to act on exogenously generated 3’P-ends. Archaeal RtcB from Pyrobaculum aerophilum does not utilise GTP to perform the ligation reaction [46]. Hydrolysis of the high-energy 2’,3’>P may instead be obligatorily coupled to the rest of the cycle, excluding 3’P-ended RNAs as alternative substrates.	

In terms of metabolic tracing, the common denominator of any RTCB-catalysed reaction is that the phosphate group at the junction between the former exonic sequences in the final RNA molecule (Fig. 1F, green “P”) originates from the 2’,3’> P rather than from GTP (Fig. 1F, blue “P”) [64]. This, together with an entirely distinct fold of the enzyme, sets RTCB-mediated RNA ligation reactions apart from the other RNA ligases, as well as from the human LIG-family DNA ligases, where the phosphoryl moiety comes from the energy-rich ATP molecule rather than the nucleic acid substrate [8, 57, 66–68]. Fascinatingly, defining features in RTCB enzymology: substrate specificity [69] and the origin of the splice junction phosphate [70, 71], alongside with the beneficial effects of magnesium and ATP (see below, also sections Sect. 5 and 6) [71], have been uncovered in HeLa extract decades before the identification of RTCB as the responsible catalyst.

Besides the conservation of the catalytic histidine and the metal binding cysteine residues, the critical residues involved in RNA binding (particularly those interacting with phosphodiester bridges) also appear widely conserved, as recently demonstrated through the comparison between archaeal and piscine enzymes [72].

Throughout the catalytic cycle of RNA ligation reactions, RTCB catalyses multiple nucleophilic attacks by the nitrogen or oxygen atoms onto the phosphoryl groups of the high-energy intermediates. To achieve this, RTCB molecules invariably employ divalent metal cations (Fig. 1F, grey spheres; see Sect. 5.2), attracting the electrons from the corresponding oxygen atoms and ultimately turning the targeted phosphorus more electrophilic [61]. Binuclear metal ion sites, where two metal ions are bound to a conserved cysteine residue, thus play a universal role in RTCB biology [20, 73, 74]. The critical cysteine (C122 in human RTCB) has been described as “catalytic” due to the loss of enzymatic activity upon its replacement [52], although it supports the reaction indirectly via the metal binding rather than through direct interaction with the substrates. Mn^2+^ ions support universally, if not exclusively, ligation activity in vitro, although the roles of metal ions in the biology of metazoan RTCB appear more complex (see Sect. 5) [61, 74, 75].

The reliance on a metal-binding cysteine residue is the Achilles heel of RTCB. Exposure to oxidising metal ions and reactive oxygen species, or even atmospheric oxygen, leads to cysteine oxidation, loss of catalytic metal binding and a concomitant abrogation of the catalytic activity (see Sect. 7) [31, 33, 76]. This may not have been critical for the ancestral RtcB molecules originating from the early anaerobic epochs (prior to the great oxygenation event during the Earth’s paleoproterozoic era) or their anaerobic descendants, but has become a liability in modern aerobic life. The half-life of RtcB/RTCB thus likely depends on the oxygen levels in the environment of the corresponding organism, the levels of oxidative stress each cell faces at a given time point and the mechanisms the species have employed to provide it with antioxidative protection.

Taken together, we have outlined the common denominators of RTCB/RtcB-catalysed reactions: endergonic RNA ligation coupled with GTP hydrolysis dependent on a catalytic histidine residue, reliance on metal ions coordinated by a critical cysteine residue, common modes of RNA binding across phyla and, ultimately, acting on 2’,3’> P and 5’OH at 3’ RNA and 5’ RNA termini, respectively. This last feature in particular allows for a potential substrate-level regulation, the first pathway-tuning mechanism to be discussed in this review.

## Chemically specific RNA termini and the substrate-level regulation

RNA termini, more precisely 3’-ends decorated with 2’,3’> P and 5’-ends with 5’OH groups are the key specificity determinants for RTCB-mediated RNA ligation. Any chemical reaction that would act on such RNA termini would affect the amounts of substrate available for RTCB, and hence indirectly the rate of the product generation.

**Table:**
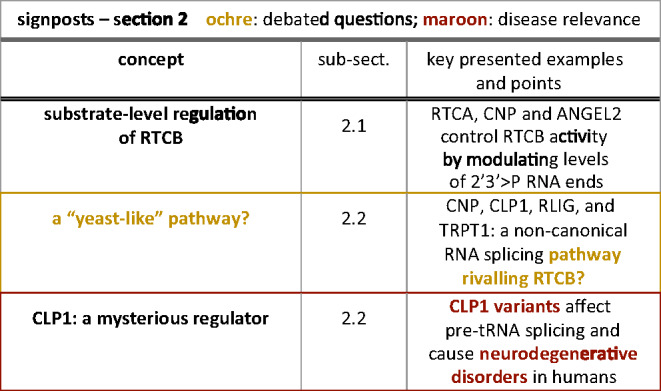

### Who snatches the cycle first? The alternative fates of 2’,3’>P RNA ends

Three enzymes in mammals have so far been observed to be able to modify the rates of RTCB-dependent reactions by modulating the availability of 2’,3’>P ends: RTCA [69], CNP [77] and ANGEL2 [78].

**Fig. 2:**
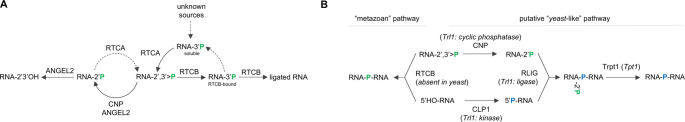
Substrate-level regulation of RTCB-mediated RNA splicing and the functional equivalence of regulatory proteins in animals with the components of the yeast pre-tRNA splicing pathway (*italic*). (**A**) (left) CNP and RTCA perform a seemingly futile cycle that may regulate the levels of 2’,3’>P-ended RNA. This activity of RTCA is however low (dotted line). (bottom) ANGEL2 hydrolyses the 2’,3’>P to non-phosphorylated product, 2’,3’OH. (middle) In its primary activity, RTCA uses 3’P-ended RNA to generate 2’,3’> P. Such 3’P may originate due to the potential inadvertent dissociation of the RTCB-bound intermediate (dashed lined). However, other, currently unknown sources of 3’P-ended RNA cannot be ruled out. (**B**) The canonical metazoan pathway (left) and the putative “yeast-like” pre-tRNA splicing pathway (right). In yeast, three enzymatic activities catalysed by distinct metazoan enzymes are performed by distinct enzymatic modules of the Trl1 protein, while the fourth reaction is catalysed by a separate protein, Trpt1. Yeast enzymatic modules functionally equivalent to the metazoan enzymes are written in *italic*. Green and blue P denote phosphoryl groups originating from the RNA substrate and GTP, respectively, illustrating different origin of the juncture phosphoryl group in the two pathways. However, there is so far no evidence that such pathway operates in full in metazoan cells, rather delegating its individual components to regulatory roles or roles in other potential pathways

2’,3’-cyclic nucleotide 3’-phosphodiesterase (CNP) and RNA 3’-terminal phosphate cyclase (RTCA) form a seemingly futile cycle of hydrolysis and regeneration of 2’,3’>P (Fig. 2A, middle-left) [79, 80]. RtcA, often described as “*de novo*” generator of 2’,3’>P (denoting 2’,3’>P generation on an already existing RNA end rather than a product of an endo- or exonucleolytic cleavage) is an ancient enzyme sharing the operon with RtcB in some bacteria, thus implying a widely conserved potential regulatory role [14, 81]. This seeming interplay extends to animals, where RTCB and RTCA co-localise in transport granules in neurons [82].

The most suitable substrate of RTCA is however not 2’P-ended, but rather 3’P-ended RNA (see Fig. 2A, middle-right). While reaction rates with the two substrates differ by five orders of magnitude despite similar enzyme-substrate affinities [79], the absolute conversion kinetics would depend on the relative local concentration of each RNA end. Creation of 2’,3’>P from 3’P by RTCA may be beneficial to rescue 3’P intermediates aberrantly dissociated from RTCB in the hypothetical condition where RTCB cannot directly re-bind the 3’P-ended RNAs (see Sect. 1.3 and Fig. 1F, bottom). Alternatively, 3’P-ended RNA may be generated though other, still elusive enzymatic activities (Fig. 2A, middle-top).

In line with its potential regulatory role, overexpression and silencing of RTCA in mouse cells was observed to increase and decrease the rates of RTCB-mediated *Xbp1* mRNA splicing, presumably through elevation and reduction of 2’,3’> P levels, respectively. CNP displays exactly the opposite effect [80].

The most recently described enzyme that affects RTCB activity at substrate level is ANGEL2 [78]. ANGEL2 displays 2’,3’ cyclic phosphatase activity, thus removing 2’,3’>P via a 2’P intermediate. Unlike CNP, ANGEL2 is not a part of an equivalent futile cycle, since it rapidly continues the reaction to generate a RNA-2’,3’OH product (Fig. 2A, left). ANGEL2 is a member of the deadenylase family, together with four human paralogues: ANGEL1, nocturnin, CNOT6 and CNOT6L. However, a unique active side loop of ANGEL2 causes a steric occlusion and thus a shift in the positioning of the 3′-terminal nucleotide within the substrate binding pocket, making ANGEL2 inactive for deadenylation but accessible towards 2’,3’>P ended RNA molecules. As an enzyme that removes 2’,3’>P, overexpression of ANGEL2 decreases 2’,3’>P levels and RTCB-mediated ligation in a manner comparable to CNP, while its silencing enhances the reaction [78].

**Table:** 

ANGEL2 in mitochondria	
Among the enzymes discussed in this review, ANGEL2 displays a unique peculiarity: a prominent population localised in mitochondria. There, it participates in non-canonical processing of mitochondrial gene junctions and is critical for functioning of the respiratory chain [28]. How distribution of ANGEL2 is controlled to fulfil these distinct roles remains unknown.	

### One substrate, two pathways: competition or regulation?

It is interesting from an evolutionary perspective that one of the three enzymes described above, CNP, is functionally equivalent with and can complement the loss of the cyclic phosphodiesterase module of the RNA ligase Trl1 in yeast (Fig. 2B) [84]. In fact, all components of the yeast pathway, including all three Trl1 modules have functional equivalents in individual mammalian enzymes. This implies that an entire alternative “yeast-like” tRNA ligation pathway (Fig. 2B, right), initiated upon CNP-mediated hydrolysis of 2’,3’>P, could theoretically compete with the canonical metazoan RTCB-mediated reaction (Fig. 2B, left). The other members of such conceivable yeast-like pathway are RNA kinase CLP1, RNA ligase RLIG1 and RNA phosphotransferase Trpt1.

CLP1 is a 5’ kinase that phosphorylates the 5’OH group at the 5’ RNA end [85]. Similarly to the removal of 2’,3’>P by CNP on the 3’ end, phosphorylation of 5’OH-end eliminates the substrate for RTCB-mediated ligation (Fig. 2B, middle).

The functions of CLP1 in the context of RNA splicing however remain poorly understood. A single amino acid replacement in the Walker A motif K127A, which diminishes ATP binding, as well as the pathogenic replacement R140H were shown to cause alterations in pre-tRNA splicing in mouse models [33, 86] and patient-derived iNeurons [87].

Critically, CLP1 interacts with tRNA splicing endonuclease (TSEN) complex [23], which precedes RTCB, generating the suitable ends for the ligation reaction (see Fig. 1B). Single amino acid replacements in all TSEN subunits, as well as in CLP1, result in neuronal disorders in humans [88] [reviewed in [89]]. The RNA ends are however estimated to be out of reach for CLP1 while bound within TSEN, implying that tRNA exons would first need to dissociate before binding CLP1 [90, 91]. Recent in vitro reconstitution studies demonstrated that CLP1 does not affect TSEN activity in the purified system [92, 93]. Nevertheless, CLP1 appears to be a negative regulator of RTCB in insect cells, as observed through increased RNA ligation upon CLP1 silencing, assessed through tricRNA formation (see Fig. 1B) [92]. This is in line with RNA phosphorylation by CLP1 being a competing pathway to its ligation by RTCB.

The described consequences of (1) the exclusion of CLP1 from the in vitro reconstituted system (no effect), (2) its silencing in insect cells (boosted intron ligation) and (3) the expression of loss-of-function variants in mice (accumulation of non-ligated tRNA fragments) thus do not appear fully aligned. CLP1 biology becomes further complex due to its separate function in regulating transcriptional termination via association with the polyadenylation machinery [86, 94–96]. Its silencing thus causes potential pleiotropic effects. To address the gaps described above, a more detailed biochemical characterisation of the pathogenic CLP1 variants and their effects on TSEN stability, RNA phosphorylation and mRNA 3’end formation will be required.

The most recently discovered enzyme functionally analogous to the activities operating in the yeast pathway is the RNA ligase RLIG1 (C12orf29) [66]. Together with the above described CNP and CLP1, as well as the phosphotransferase Trpt1 [97] which transfers the 2’P group from the RNA product to NAD^+^[98], RLIG1 would be able to perform an alternative “yeast-like” RNA ligation pathway in humans (Fig. 2B, right). However, this does not appear to occur in cultured HeLa or HEK cells, where RLIG1 does not seem to contribute to tRNA exon sealing in vivo [66]. “Yeast-like” pathway may however operate in specific cell types or conditions, or in cleavage-ligation reactions other than pre-tRNA splicing. This is supported by the nearly exclusive expression of Trpt1 mRNA in heart and skeletal muscle – tissues particularly exposed to oxidative burden [97], as well as with the observation that silencing of RTCB or associated components does not quantitatively abrogate UPR under ER stress conditions [99].

For example, in what appears to be a gender-specific role in female mice, RLIG1 seems to act in tRNA metabolism at the level of tRNA repair [66]. It is important to note that RLIG1 does not directly compete with RTCB for the substrate, as it acts on RNA ends with 5’P and 3’OH chemistry; furthermore, it depends on ATP rather than GTP [66]. How and when RTCB and RLIG1 pathways intertwine is thus yet to be established.

Together, this chapter described three enzymes – CNP, RTCA, and ANGEL2 – that may modulate RTCB-mediated RNA ligation reactions by acting on 2’,3’> P-decorated 3’ends, and CLP1, which acts on 5’OH ends. However, most of these effects are observed upon artificial modulation of expression of the corresponding enzymes. The conditions where cells endogenously modulate RTCB-mediated reactions through the levels or activities of the described proteins hence need to be determined.

## Co-purification meets co-evolution: Uncovering the layers of the tRNA-LC

The complexities of eukaryal cells, including compartmentalisation, often aerobic metabolism, and the need for fine-tuned regulation necessitated adaptations that RTCB alone could not provide. For these purposes, RTCB binds to several factors with still not fully understood roles. Not all of these proteins were discovered simultaneously, nor bind RTCB at the same time. This section will provide the overview of evolution of our understanding of the non-canonical RNA splicing in animals, and introduce the layered complexity of what is now referred to as the tRNA-ligase complex (tRNA-LC): a dynamic, multiprotein complex highly adapted to sustain non-conventional RNA splicing reactions in animals.

**Table:** 

signposts – Sect. 3		
concept	sub-sect.	key presented examples and points
“canonical” tRNA-LC	3.1	RTCB, DDX1, CGI-99, FAM98B and Ashwin co-purified as the “canonical” tRNA-LC
“core” tRNA-LC	3.2	RTCB, DDX1, CGI-99 and FAM98B are mutually dependent within the “core” tRNA-LC
FAM98 paralogues	3.3	a homology-based search identifies A- and C- paralogues of the “canonical” FAM98B subunit (see Sect. 4)
co-evolution as a tool	3.4	monitoring co-evolutionary patterns to reveal new complex members
“expanded” tRNA-LC	3.4	co-evolution identifies Archease (see Sect. 5) and PYROXD1 (see Sect. 7)

### Sticking together: co-purification of the “canonical” tRNA-LC subunits

Despite early indications of a functional yeast-like RNA splicing systems in animals (see Sect. 2), RTCB, then referred to as HSPC117, emerged as the essential mammalian pre-tRNA splicing enzyme [52]. This finding came in parallel with the discovery of RtcB as the RNA ligase in *Bacteria* [100] and in *Archaea* [60]. The identity of RTCB as the metazoan RNA ligation enzyme was originally revealed through activity-guided purification. In contrast to the bacterial and archaeal homologues, in both activity-guided purification and subsequent immunoprecipitations RTCB co-purified with four additional proteins, identified as DDX1, CGI-99 (RTRAF/hCLE), FAM98B and Ashwin [52].

DDX1, CGI-99, FAM98B and RTCB had been previously detected together in protein:RNA granules in neurons, which also contain RTCA (see Sect. 2) [82]. Separately, DDX1, CGI-99 and RTCB were found to form an apparent trimeric complex binding to serum response elements in promoters [101]. DDX1 had been known for the role during HIV infection [102, 103] and double stranded break repair [104]. In turn, CGI-99 had been reported to interact with influenza polymerase [105] and RNA polymerase II [106], as well as with ninein, blocking its phosphorylation in vitro [107]. The key finding leading to the concept of a stable and well-defined RTCB-containing complex, initially named “particle X”, was an observation that all five subunits co-purified with spliceosomal factor 3b [doctoral Thesis: [108]; published in [52]. However, none of the proteins had been linked with RNA splicing prior to identification of RTCB as the mammalian RNA ligase [52].

**Fig. 3:**
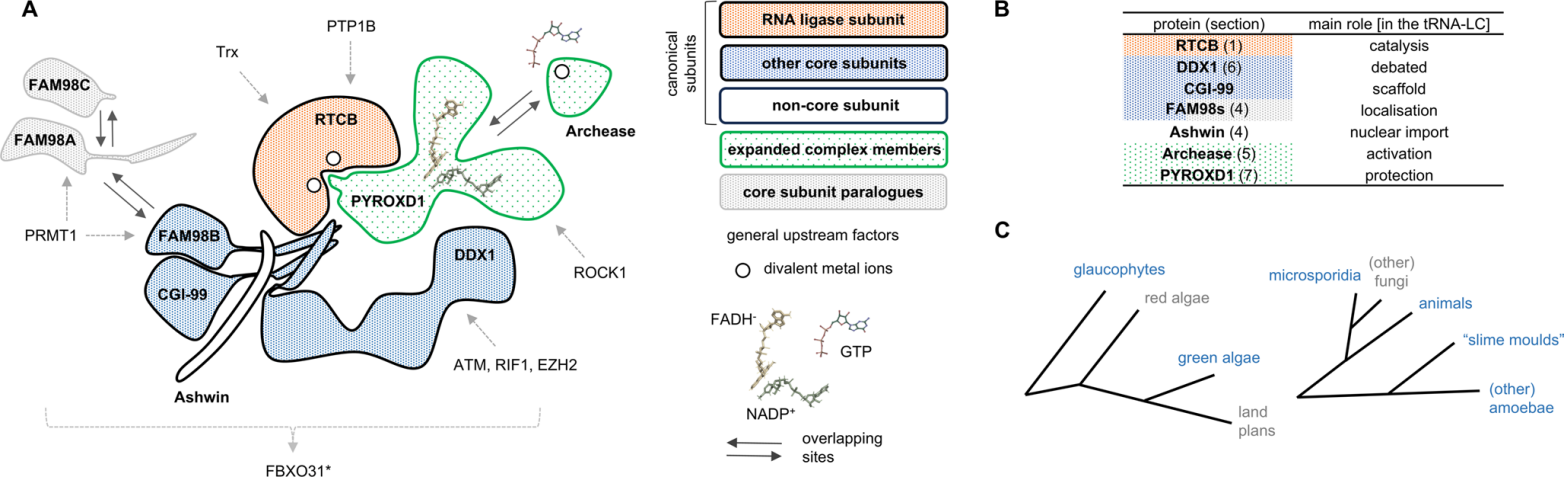
The layered complexity of the expanded tRNA-LC and its phylogenetic distribution. (**A**) The components of the tRNA-LC are grouped and colour-coded according to the chronological sequence and the tools use for their discoveries. RTCB (orange), also individually present in *E. coli*, is joined by DDX1, CGI-99 and FAM98B (blue) to form the “core” complex in animals, which can associate with the fifth initially identified (canonical), but non-core subunit Ashwin (white). PYROXD1 (green) is bound to the tRNA-LC between catalytic cycles. The central image displays the potentially most abundant form of the tRNA-LC: the nuclear form (containing FAM98B) in the “resting”, PYROXD1-bound state. (top right) Archease (green) transiently replaces PYROXD1 to catalyse RTCB guanylylation, while (left) FAM98A isoform (grey) can replace FAM98B in the cytoplasm; less abundant FAM98C is also detected. For simplicity, Archease is displayed as a monomer and with a metal ion pre-bound, as it appears in RTCB: Archease complex with 1:1 stoichiometry and containing a Archease-bound metal ion. Whether Archease may form homodimers in solution and if it brings a pre-bound metal to the RTCB: Archease complex is yet to be dissected. The more general regulatory proteins (grey arrows) are described in the text. *FBXO31 only targets FAM98A- and not FAM98B-containing tRNA-LC. (**B**) An overview of the main functions the expanded tRNA-LC subunits are exerting within the complex; for more details, as well as the functions (potentially) unrelated to the tRNA-LC, see the indicated sections. (**C**) Exemplary clades of *Eukarya* within *Archaeplastida* (top) and *Opisthokonta*-*Amoebozoa* groups (bottom) where RTCB is absent (grey) or present in at least some species (blue). Such peculiar and overlapping phylogenetic distribution of all the subunits of the expanded tRNA-LC subunits allowed identification of Archease and PYROXD1, which artefactually dissociates during (co)-purification due to dilution and oxidation of the critical cofactor NADPH

At the time of this discovery, the complex consisting of the five above listed proteins (RTCB, DDX1, CGI-99, FAM98B and Ashwin) was named the tRNA-ligase complex [52], later abbreviated as the tRNA-LC [31, 109]. Here, the five originally described complex members will be referred to as the *canonical subunits* of the tRNA-LC (Fig. 3A, black outline; see below and Fig. 3B for the simplified guide to the functions of each tRNA-LC subunit). However, already the initial observation revealed that not all subunits were “equal”, and hinted at what is shaping into a two-tier tRNA-LC where some subunits appear more critical for the stability of the complex than the others.

### A core in a shell: the hierarchical layers of the tRNA-LC

While RTCB co-purified with the four additional proteins described above, two of them stood out in the initial analysis: Ashwin and RTCB. Namely, silencing experiments revealed FAM98B to co-deplete with CGI-99 and DDX1, and vice versa, DDX1 was co-depleting with FAM98B and CGI-99. Yet, removal of Ashwin did not affect the steady state stability of any of the other subunits, and RTCB remained independent, in terms of stability, from the rest [52]. More modern and efficient, degron tag-based protein clearance technology revealed RTCB to also rely on the other subunits for stability, albeit only partially [110]. Reliance on the other core subunits has particularly important consequences for DDX1, which performs functions apparently not linked to the tRNA-LC (see Sect. 6). Dependency of DDX1 on the other subunits implies that either the entire tRNA-LC is participating in DDX1-associated processes, or that they are carried out by an independent, short-lived, high turnover pool. Further studies will be needed to test these hypotheses.

These early observations already painted a multi-tier tRNA-LC [52]. At the core, there is RTCB, the catalytic subunit remaining largely stable without its co-purifying partners (Fig. 3A, orange). This is in line with RtcB presumably operating without the other canonical complex members in *Bacteria* and *Archaea*, where none of their direct orthologues were reported nor linked with RNA splicing [58]. Next come CGI-99, FAM98B and DDX1, which seem to be critically dependent on each other for stability. RTCB, together with those three proteins, forms what has been referred to as the “*core*” tRNA-LC [74] (Fig. 3A, blue). Seemingly redundant Ashwin may optionally associate with those core subunits without affecting their stability (Fig. 3A, white).

The hierarchy within the metazoan tRNA-LC is in line with its latest reconstitution [74] and its first published structures [72, 111]. Namely, both the cross-linking mass spectrometry experiments of the human core tRNA-LC and the cryo-electron microscopy model of a truncated full complex reveal that CGI-99 and FAM98B form a dimer that associates with RTCB and the C-terminal helix of DDX1 across an extensive interface encompassing all four proteins. This helix appears to be a unique identifying feature of eukaryal DDX1 and no homologous sequences can be identified through simple blast of other genomes.

Together, these data paint a relatively simple picture of the *canonical* complex where RTCB constitutively associates with three core co-dependent subunits, and ultimately optionally with Ashwin. However, recent data reveal that neither the canonical subunits are always present, nor are these five proteins sufficient for RTCB to perform and sustain its functions.

### Not invariable after all: paralogues of FAM98B

FAM98B was identified as a canonical and core subunit of the tRNA-LC. However, analysis of stress granules [112], as well as viral ribonucleoprotein particles [113], could not detect FAM98B, but rather its paralogue FAM98A (Fig. 3A, grey). Recent findings showed a more complex picture, where any of the three FAM98 paralogues (A, B or C) can individually incorporate into the tRNA-LC, in a process determining whether Ashwin would ultimately join the complex [110, 111]. These findings, discussed in Sect. 4, revealed that plasticity exists even among the core subunits, bringing up additional qualities to the tRNA-LC, in this case regulation of the nuclear import (see Sect. 4). FAM98A and C can thus, despite not being identified as canonical subunits of the RTCB-based tRNA-LC, replace FAM98B in the core complex.

### Coming and going: the patchy evolution of the expanded tRNA-LC

While co-purification gave rise to the canonical tRNA-LC, enriched through identification of FAM98 paralogues, it will ultimately have been co-evolution that revealed the complete picture and identified further essential members. Namely, the analysis of orthologous groups, which identifies orthologues of a selected protein across many species, and then ranks other proteins according to the degree of overlapping distribution patterns, has been strikingly successful for the identification of new subunits critical for the tRNA-LC in animals.

The reason for this is a uniquely patchy evolution of RTCB. Arising in ancient anaerobic times, RTCB became a liability for the aerobic organisms, likely due to its sensitive metal site [31]. Those organisms had a stark choice: not to rely on RTCB for unconventional RNA ligation and replace it with another RNA ligase, or to develop mechanisms to safeguard its function (see Sect. 7). Surprisingly, such decisions seemed to have happened many times across evolution [52].

For example, RTCB has been entirely lost in land plants, yet closely related green algae have retained the enzyme [114]; the puzzle does not end there, as red algae again display loss of RTCB, while their *Glaucophyte* relatives retained it (Fig. 3C, left). Similarly, RTCB is present across animals, as well as largely parasitic microsporidian fungi [115], yet absent from the major fungal phyla, to ultimately re-appear in *Amoebozoa*, including amoebae [116] and “slime mould” *Dictyostelium* [117] (Fig. 3C, right). Similarly patchy patterns spread further across eukaryal species. These patterns currently appear stochastic and too complex to be presented here in full; which specific conditions led different clades to opt for retaining RTCB or replacing it remains mysterious and should be a subject of future research. Besides the detailed literature research, such patterns can be easily obtained from a simple blast of distinct RTCB amino acid sequences against the proteomes of phyla of interest.

**Table:** 

One species, three RTCBs	
While the phylogenetic analysis posits whether a protein, e.g. RTCB is presents or not in a given genome in a binary manner, recent findings highlight the possibilities of multiple RTCBs within a single organism. Diplonemids, a group related to euglenas, encode for three distinct RTCB genes [184]. Peculiarly, at least one of these proteins is mitochondrial, where it likely plays a role in “stitching” mitochondrial genes from individually encoded fragments, presenting an entirely new role of RTCB [63]. Finally, the three homologues appear to have been acquired separately from bacteria, providing a clue that the repeated horizontal gene transfers across phyla may be the cause of the patchy phylogeny of the tRNA-LC.	

This seemingly unpredictable phylogenetic distribution, besides the puzzling evolutionary questions, offers a remarkable tool: if mechanisms and factors have evolved with RTCB to sustain it in aerobic environment, the enzyme may not appear and disappear in different species alone, but together with its associated entourage.

Taking advantage of this logic has borne fruit twice: in discovering Archease [58] and later, PYROXD1 [31]. Both enzymes are essential for RTCB activity in animals, even more so than the canonical subunits. Both of them also interact with RTCB, mutually exclusively occupying its active site during and between the reaction cycles, respectively [75, 120]. While Archease potentially operates on touch-and-go basis during the catalytic cycle (see Sect. 5), PYROXD1 interaction is more stable and forms the “resting state” of the tRNA-LC between ligation reactions (see Sect. 7). However, PYROXD1 was not identified among the canonical, co-purified subunits because NADPH, a small molecule on which the PYROXD1:RTCB interaction relies, is diluted out and oxidised during purification or immunoprecipitation procedures [31].

These examples illustrate how the knowledge of RTCB evolved from a set of canonical subunits based on co-purification, to encompass additional critical proteins discovered due to evolutionary peculiarities. We will here refer to the full set of proteins as expanded tRNA-LC, and to Archease and PYROXD1 as *expanded* complex members (Fig. 3A, green).

The next chapters will individually dissect how each key subunit of the expanded tRNA-LC contributes to its biology in animals, in concert with a series of multiple universal regulatory cellular proteins, here described as additional *general upstream factors* (Fig. 3A, dashed grey arrows) that affect their activity and localisation. Multiple subunits have additional reported functions, but it is often unclear if these involve the entire complex and depend on its catalytic activity. We’ll briefly address these before focusing on their role in non-canonical RNA splicing.

## Trafficking of the tRNA-LC

Eukaryal tRNA-LC operates in two different, membrane-segregated compartments: nucleus for pre-tRNA splicing and cytoplasm for *Xbp1* mRNA splicing during the unfolded protein response (UPR). How RTCB is trafficked from one compartment to another has until recently remained mysterious.

**Table:**
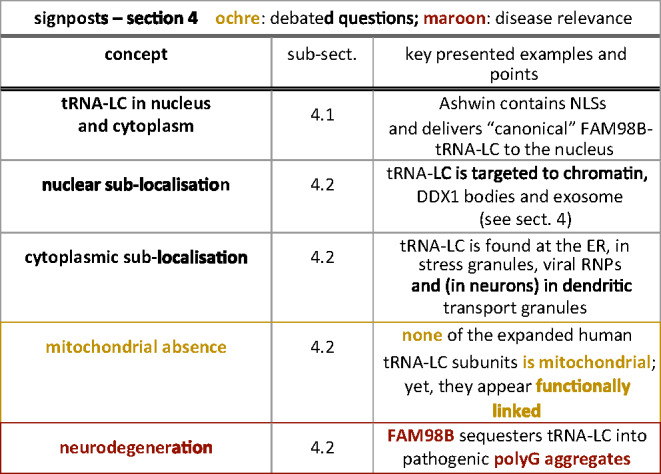

### tRNA-LC between cytoplasm and nucleus: FAM98B-Ashwin carrier

This changed with a recent breakthrough. Removal of Ashwin was observed to result in the accumulation of pre-tRNA fragments, a hallmark of deficiency in the ligation step during pre-tRNA splicing [110]. Yet, as a non-core subunit, silencing of Ashwin was not observed to impair tRNA ligase activity in cell extracts [52], and Ashwin is not required for RNA ligation by the recombinant tRNA-LC in vitro [74]. This prompted the speculation that the absence of Ashwin may abrogate the nuclear localisation of the tRNA-LC.

In subsequent experiments it was demonstrated that Ashwin is indeed required for the nuclear import of the tRNA-LC due to the presence of two distinct and in part mutually redundant nuclear localisation signals [110]. More strikingly, Ashwin only binds to the complex loaded with the canonical FAM98 paralogue, FAM98B. Although Ashwin mediates nuclear import of the tRNA-LC, a small population of FAM98B-containing complex is found in cytoplasm, presumably due to sub-stoichiometric amounts of Ashwin. In contrast, the entire amount of the complex loaded with FAM98A stays in the cytoplasm. Just like Ashwin, removal of FAM98B leads to accumulation of pre-tRNA fragments. Importantly, pre-tRNA fragment accumulation in Ashwin-depleted cells can be rescued by artificial nuclear targeting of the tRNA-LC through addition of the nuclear localisation signal onto RTCB. The third paralogue, FAM98C can also traffic the tRNA-LC to the nucleus, yet its contribution is limited [110], likely due to its lower expression levels [121].

**Fig. 4:**
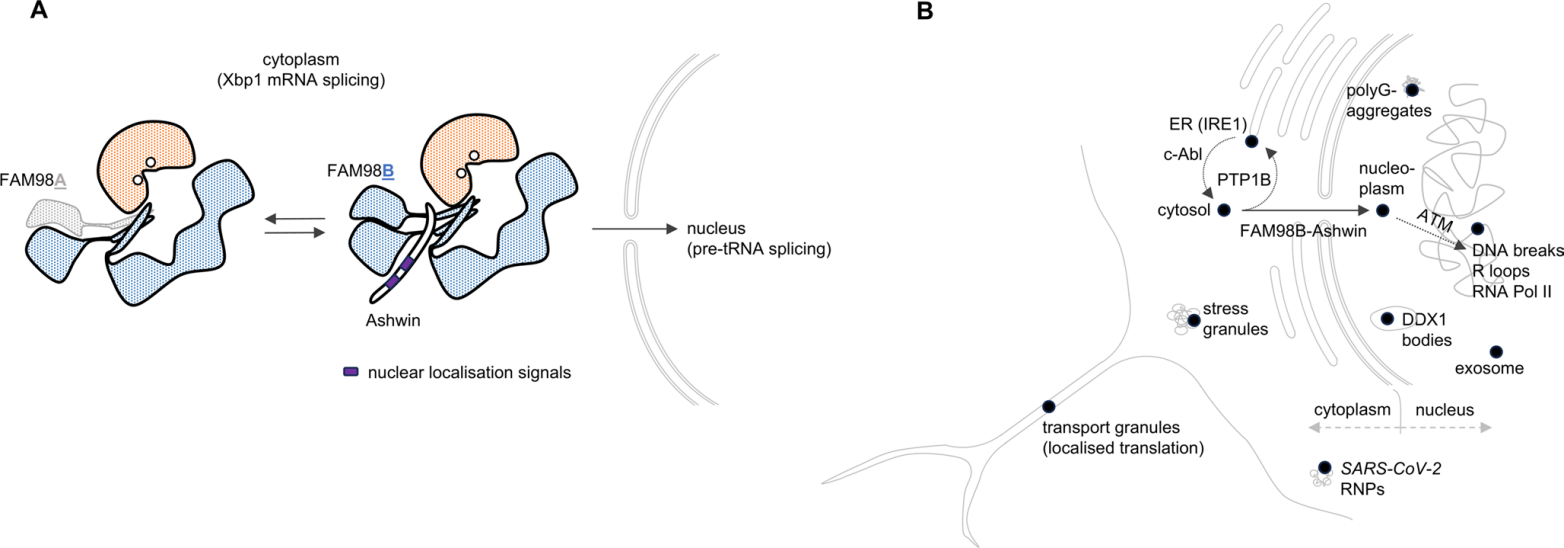
Nucleo-cytoplasmic trafficking of the tRNA-LC subunits through the FAM98B-Ashwin axis and the localised sub-distribution of the tRNA-LC subunits within these compartments. (**A**) The cytoplasmic tRNA-LC predominantly exists in two forms, containing FAM98A or FAM98B paralogues; the paralogue B recruits Ashwin, which traffics the tRNA-LC to the nucleus using two mutually redundant nuclear localization signals (violet). (**B**) At least one subunit of the tRNA-LC was demonstrated to reside at each displayed location (•), and only a selection of reported localisations and targeting mechanisms is shown. ER localisation is at least in part regulated by RTCB (de)phosphorylation and IRE1 interaction. Studies on DNA breaks and R-loops were primarily based on DDX1, while those on Pol II on CGI-99. FAM98A and B were reported to drive localisation to stress granules and poly-G aggregates, respectively. Mutual co-dependency of the core subunits suggests that the tRNA-LC, rather than the individual subunits localise to the designated structures. Of note, while the neuron was displayed to include dendrite-localised translation, the majority of observations were made in immortal cell lines such as HEK293

This finding demystified the need for multiple pools of the core tRNA-LC, where the FAM98 paralogues act as gateways: while the canonical paralogue B binds to Ashwin and traffics the complex to the nucleus (Fig. 4A), the paralogue A is bound within the cytoplasmic complex.

### Sub-distribution of the tRNA-LC: ER, RNA granules, PolyG aggregates, chromatin…

The localisation of the tRNA-LC is not restricted to nucleus vs. cytoplasm resolution (Fig. 4B). For example, the FAM98B-containing tRNA-LC binds RNA polymerase II within the nucleus, affecting mRNA transcription rates through as of yet unknown mechanisms [122]. The same complex was observed in the cytoplasm and has the ability to bind mRNA caps; silencing of CGI-99 (referred to as hCLE) decreases protein translation [123], though the direct link with the mRNA cap binding is not yet demonstrated.

On the other hand, DDX1 was reported to perform various nuclear functions associated with chromatin [104, 124], RNA exosome (a molecular machine tasked with clearance of RNA molecules within cells) [125], and the eponymous DDX1 bodies [126, 127]. In the cytoplasm, DDX1 was observed in stress granules [112] and, in dendrites of neurons, in transport granules used for localised protein translation [82]. Together with FAM98A and RTCB, DDX1 was detected in viral ribonucleoprotein particles [128] (see Sect. 6). The mechanism trafficking the tRNA-LC complex to the nucleus via Ashwin would thus have a critical effect on all these sub-populations and their respective functions.

Besides their roles in nucleo-cytoplasmic trafficking, FAM98 proteins also govern sub-localisation of the tRNA-LC under specific conditions.

FAM98A localises to various cytoplasmic stress granules, which was attributed to its long, unstructured C-terminal region [112]. It is unknown how sequestration of FAM98A during oxidative stress interplays with oxidative inactivation of RTCB (see Sect. 7). The unstructured region of FAM98A is also targeted for methylation mediated by PRMT1, an arginine methyltransferase that acts on RG-rich repeats [129]. How such methylation affects the localisation of the tRNA-LC is yet to be investigated.

On the other hand, recent finding placed FAM98B, and in particular its disordered tail, under the spotlight from an entirely new angle [109]. Several neurodegenerative disorders such as fragile X-associated tremor/ataxia syndrome (FXTAS) or neuronal intranuclear inclusion disease (NIID) are characterised by the nuclear accumulation of polyglycine aggregates. Such polyglycine (polyG) peptides result from pathogenic, genomic multiplication of GGC repeats in normally untranslated regions of other genes [reviewed in [130, 131]. While GGC repeat/polyG-disorders represent a case where a causative relationship between the aggregate formation and disease phenotype has been successfully established [132, 133], the mechanism of their pathogenicity remains unknown. Recent findings revealed that pathogenic polyG aggregates can sequester otherwise “healthy” proteins that inherently contain glycine-rich regions, and remarkably, the tail of FAM98B is the most glycine-rich protein sequence in humans [109]. Sequestration of FAM98B into polyG aggregates concomitantly co-sequesters the tRNA-LC, resulting in disrupted pre-tRNA splicing [109], implicating erroneous tRNA processing as a potential cause and hallmark of polyG disorders. Whether FAM98B is specifically directed towards polyG aggregates, and FAM98A to the stress granules, and which potential differences between FAM98A- and B-containing tRNA-LC pools beyond diverging nucleo-cytoplasmic distribution drive such distinct fates remains unknown.

Another layer of sub-localisation of the tRNA-LC is linked to its role in the UPR. tRNA-LC subunits have been observed in the microsomes and in the immunoprecipitates of IRE1 [134, 135], the ER-residing endonuclease that initiates the splicing cascade of Xbp1 mRNA (see section 1). Tyrosine phosphorylation of RTCB by c-Abl abolishes its attachment to IRE1, which is restored via dephosphorylation through PTP1B [136]; interestingly, both c-Abl [137] and PTP1B [138] are well-known targets of redox-regulation. Yet, despite interaction with ER-residing IRE1, microscopy data imply that a large fraction of the cytoplasmic tRNA-LC remains freely diffused in the cytosol [110]. It thus remains mysterious how dephosphorylation recruits the presumably small sub-population of the tRNA-LC to the ER, whether mechanisms beyond phosphorylation mediate this recruitment, and ultimately to which extent the ER-adjacent localisation is critical for efficient *Xbp1* mRNA splicing in the first place.

**Table:** 

A peculiar non-localisation	
One of the organelles where none of the human subunits of the expanded tRNA ligase complex were identified is mitochondrion. This is particularly interesting as mitochondria are particularly affected in cells with PYROXD1 perturbations [124, 146, 148] (see section 7). What is the mechanism of this link and whether it occurs via the interplay with the core tRNA-LC is unknown. Of note, several *Euglenozoa* species, including trypanosomes [141] and diplonemids [184], contain RTCB in mitochondria, yet with likely distinct functions.	

Taken together, tRNA-LC is trafficked from cytoplasm to the nucleus via the FAM98B-Ashwin carrier. Within these compartments, it is further distributed to fulfil diverse functions. A number of aspects of such subsequent distribution to specific sub-locations remain to be tackled.

## A brief action of Archease and the cycle of RTCB guanylylation

We have described how the peculiar co-evolution of Archease with RTCB led to its discovery (Sect. 3). Due to the technical aspects surrounding the discovery of Archease, it was initially described as a “cofactor” exclusively required for the “multiple turnover” (i.e. the ability to perform more than one consecutive catalytic cycle) of the tRNA-LC, downplaying its essential catalytic role. Later, recombinant reconstitutions clearly demonstrated that not even a single turnover of the tRNA-LC was possible without its involvement: Archease is critical for guanylylation of RTCB, itself a necessary step in the catalytic cycle. Hence, Archease will be here described as the activating component of the expanded metazoan tRNA-LC rather than strictly a multiple turnover cofactor.

**Table:**
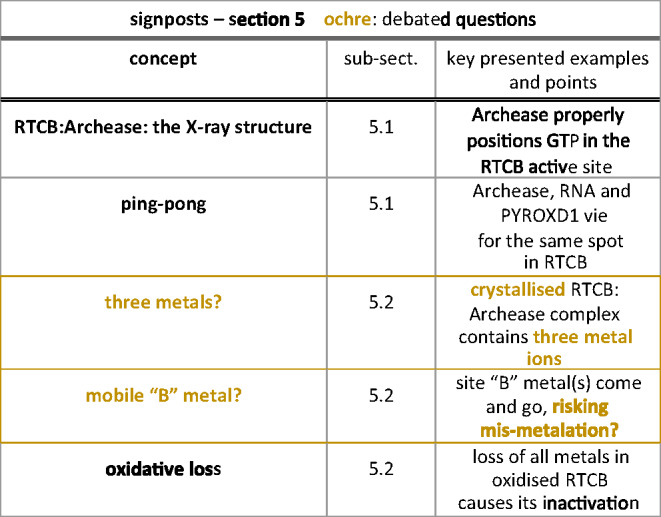

### Crystal structure of the RTCB-Archease complex and a critical role in GTP positioning

**Fig. 5:**
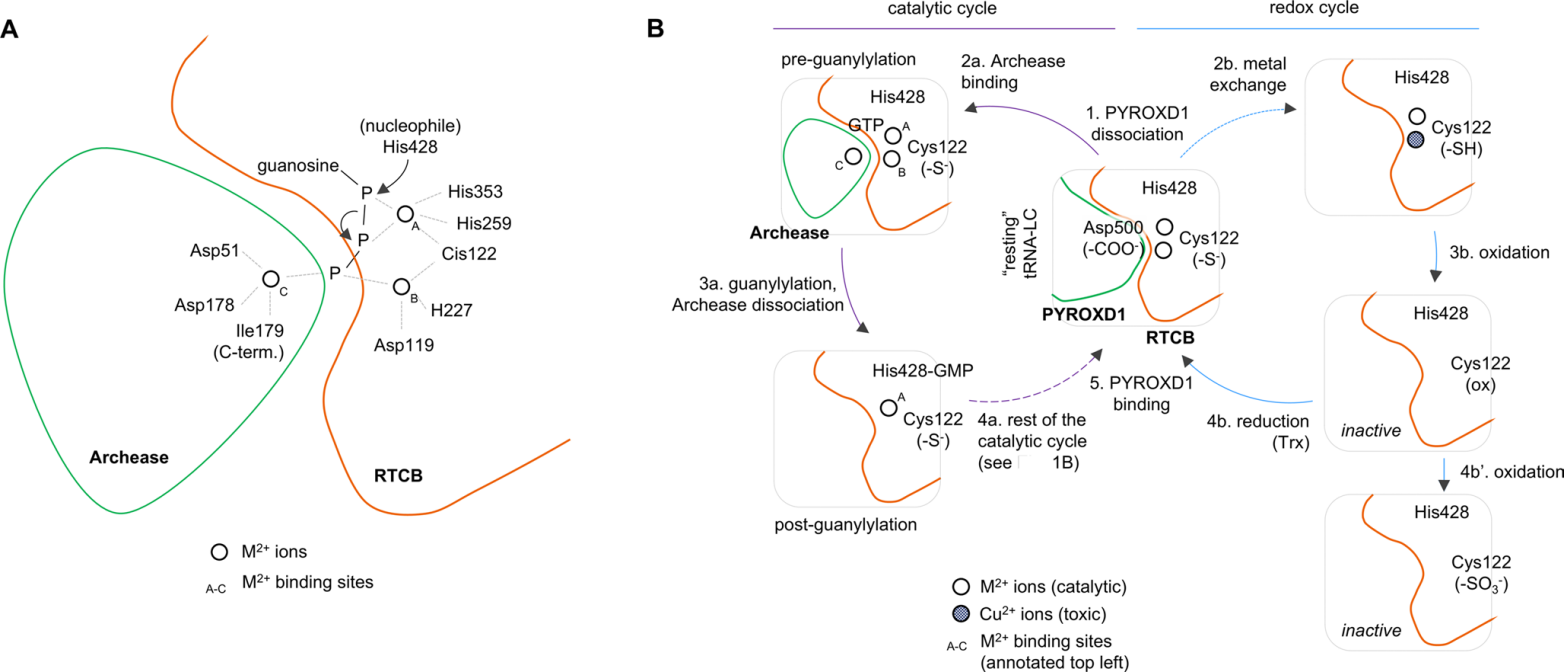
The catalytic mechanism of Archease and the metal association-dissociation cycles of RTCB. (**A**) The crystal structure of the RTCB: Archease complex revealed the critical role of Archease for RTCB guanylylation. Coordination of γ-phosphate is involved in GTP positioning and stabilisation of the leaving pyrophosphate. (**B**) Metal association-dissociation cycles are derived from biochemical experiments and several recent X-ray diffraction and cryo-EM models, in particular of the Archease: RTCB complex (top left), PYROXD1:RTCB complex (middle) and oxidised RtcB (bottom right). The catalytic cycle can only be initiated upon dissociation of PYROXD1 (1) and Archease binding (2a), resulting in the trinuclear active site (top left). Histidine guanylylation and dissociation of Archease (3a) may result in guanylylated RTCB containing only one metal ion (bottom left), which undergoes remetalation at a yet undefined stage during the rest of the catalytic cycle (4a) before PYROXD1 binds again (5). Alternatively, dissociation of PYROXD1 opens the door for the dissociation of site B metal ion and binding of Cu^2+^ (2b), which presumably triggers inactivating oxidation (3b, 4b’) of the invariant cysteine residue (right). The currently undefined, reversibly oxidised state is likely metal-free, as is the overoxidised RTCB (bottom). Thioredoxin is critical for restoring the activity of the tRNA-LC (4b) which is likely coupled with remetalation before PYROXD1 can bind again (5)

The latest breakthrough in understanding the catalytic role of Archease came from a recently reported crystal structure of the RTCB: Archease complex across two catalytic stages [22]. The key observation is that within the RTCB: Archease complex, Archease coordinates a metal ion, which in turn binds the γ-phosphate of GTP (Fig. 5A), presumably to correctly position the triphosphate group of GTP for the nucleophilic attack on the α-phosphorus, and to stabilise the resulting pyrophosphate leaving group. Of note, this is a third metal ion site, distinct from the other two metals directly bound by the cysteine residue in the RTCB active site.

The study had additional important implications. First, GTP and a divalent metal ion (most prominently Mn^2+^) are critical for binding of Archease to RTCB. This binding was only observed upon cross-linking. Cross-linking reagent was however not used for crystallisation, where the proteins are present in much higher concentrations, shifting the equilibrium towards the complex formation. The next key finding is that Archease occupies the RTCB active site, making its binding mutually exclusive to the binding of the RNA substrate. The same site is occupied in the resting state between the catalytic cycles by the other expanded tRNA-LC subunit, PYROXD1. Peculiarly, Archease-mediated RTCB guanylylation and PYROXD1 binding share another function: prevention of oxidative damage [120]. This will be discussed in more detail in Sect. 7. Finally, the structural dissection of RTCB guanylylation by Archease brought the proposed role of DDX1 under further scrutiny (see Sect. 6).

A remaining mystery is, however, why Archease is not present in all species together with RTCB/RtcB, including prominent examples such as *Escherichia coli*, and furthermore, why it is not fully critical for catalysis in some organisms where they co-exist, including the archaeon *Pyrococcus horikoshii* [143]. Understanding how Archease co-evolved with RTCB and the other components of the expanded tRNA-LC at the residue level will be needed to shed light on unique mechanistic differences in RTCB/RtcB guanylylation across domains of life.

### From three to zero, and back: the emerging RTCB (re)metalation cycles

As described above, one of the key findings from the structure of the RTCB: Archease complex is the presence of a new, third metal ion site, coordinating the GTP γ-phosphate [75]. The same publication also provided new insights on the previously known, binuclear site in the RTCB core.

In the resting state, i.e. the PYROXD1-bound state between the catalytic cycles, the tRNA-LC contains two metal ions in the active site of RTCB, coordinated by both RTCB and PYROXD1 [111, 120] (Fig. 5B, middle). RTCB critically employs the invariant cysteine residue (compare with Fig. 5A), while PYROXD1 uses its C-terminal aspartate. Metal binding site occupancy may however change during and after guanylylation. For RTCB to undergo guanylylation, PYROXD1 first needs to dissociate (step 1, see Sect. 7) to allow binding of Archease [120]. Strikingly (steps 2a and 3a), while one metal ion site (designated as A) was observed to be occupied in both pre- and post-guanylylation RTCB:Archease complexes, the other site (B) was occupied only before guanylylation (Fig. 5B, compare left top and bottom). The same site (B) appears empty in multiple, even if not all, GTP-free structures of RTCB, both from humans and archaea [20, 46, 75].

The above described structures of RTCB:Archease complexes and the structures of RTCB with varying presence of guanosine nucleotide support the notion that not all tRNA-LC metal sites are equal. Specifically, while the site A appears invariably occupied in the resting state and throughout guanylylation cycle, the occupancy of site B may be more dynamic and potentially GTP-dependent; finally, site C, composed exclusively of Archease side chains is naturally only occupied while Archease is part of the complex. Upon the completion of the catalytic cycle (step 4a), binding of both metal ions is presumably restored before re-binding of PYROXD1.

**Table:** 

Two or three metals?
It is currently not clear whether three metal ions are simultaneously required for guanylylation to occur (as depicted in 5A and 5B, top left). If so, the third metal ion (in site C) would be brought in by Archease before interacting with RTCB. Alternatively however, the metal ion from site (B) could migrate to site (C) upon binding of (initially metal-free) Archease, explaining the emptiness of site (B) post-guanylylation (Fig. 5B, bottom left) and implying that triple-metal state observed in the pre-guanylylation crystal structure (5A and 5B, top left) may not necessarily be required in solution [58]. Archease from different sources has been crystalised in both metal-free [43, 58] and metal-bound states [38]. Furthermore, the structures differ in oligomeric state: crystals containing both monomeric [38, 58] and dimeric [43] Archease in the asymmetric unit, as well as a single, monomeric NMR-based structure [200] were reported. Whether Archease forms dimers in solution, and if it pre-binds a metal ion which it subsequently brings into the Archease:RTCB complex is thus yet to be determined.
What is the identity of the metal ions?
Metal binding dynamism may shed light on varying reports regarding the metal ion requirements for RTCB-mediated catalysis. While Mn^2+^, and to a lesser extent Mg^2+^ support the activity of the human RTCB in all reports, the effects of other metals vary across species and methodologies. Harsher demetalation procedure seems to render the complex more manganese-dependent [58] compared to the softer metal chelation [98]. This implies that Mn^2+^ may be critical for site A, while other metals may also sustain the activity in the more dynamic site B. Further analyses, though experimentally challenging, are needed to determine which metals support the tRNA-LC activity when bound to each individual site, in particular within the living cells.

The dynamism and inequality of the metal sites are critical not only for catalysis, but also for redox-properties. Trace amounts of Cu^2+^ inactivate the tRNA-LC in the presence of oxidants [31]. This presumably occurs upon dissociation of the more loosely bound metal ion from site B and transient mis-metalation by Cu^2+^ (step 2b). This still needs to be directly tested.

The propensity of the active site cysteine to oxidation (steps 3b, 4b’) implies that RTCB in the inactive, oxidised state is effectively metal-free, as supported by the structure of the over-oxidised archaeal enzyme (Fig. 5B, bottom right) [73]. However, a partial reversibility of oxidative inactivation [31, 32] implies that a currently unknown reversibly oxidised thiol state (designated as Cys(ox) in Fig. 5B, middle right) could be reduced – and since the activity gets restored – remetalated (step 4b). Together, this paints a complex picture of highly dynamic metalation cycle where metals are exchanged in each oxidation-reduction cycle, and at least in site B, in each guanylylation cycle. Whether re-loading with metals is purely diffusion-limited and hence dependent on the relative amounts of their labile pools and their respective affinities, or if dedicated mechanisms exist to ensure proper (re)metalation of the tRNA-LC, remains entirely unknown.

## The DDX1 mystery

Perhaps the most extensively studied subunit of the expanded mammalian tRNA-LC is DDX1. It has been reported to act in varied processes ranging from viral infection to R-loop resolution. Nevertheless, whether DDX1 acts alone or within the tRNA-LC, and the mechanistic basis for these roles remain largely mysterious.

**Table:**
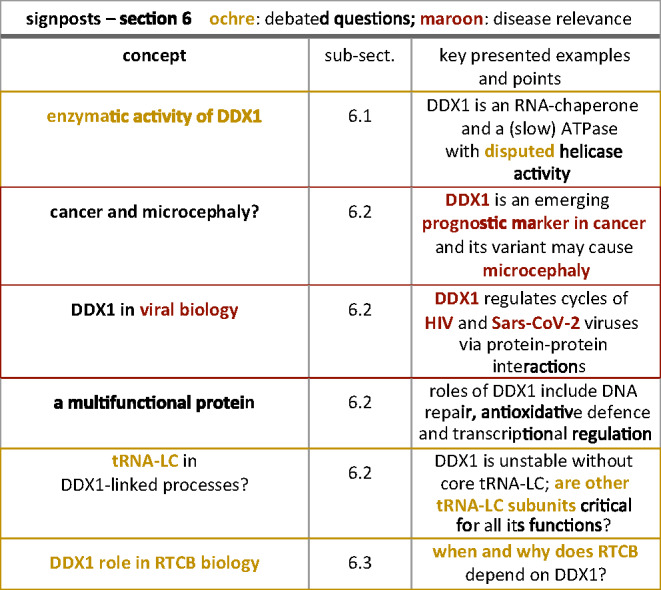

### A disputed helicase, a slow ATPase: the puzzling enzymology of DDX1

Based on homology with related DEAD box proteins, DDX1 is often referred to as RNA helicase [146, 147] (Uniprot: ATP-dependent RNA helicase DDX1). However, whether DDX1 has a *bona fide* helicase activity, suggested to be ADP-dependent and present together with RNAse activity [104] has been disputed. Such discrepancies may have been the result of the chosen expression system [148], as well as the diverging degree of purity and construct length [149, 150]. Whether the activity of co-purifying enzymes was mis-attributed to DDX1, or it harbours helicase activity dependent on other cellular components is still to be resolved. Finally, rather than being a canonical helicase, DDX1 may be an “RNA chaperone” tailored for (re)modelling of specific nucleic acid structures [151, 152], as was demonstrated with formation of RNA: DNA hybrids [147].

**Fig. 6:**
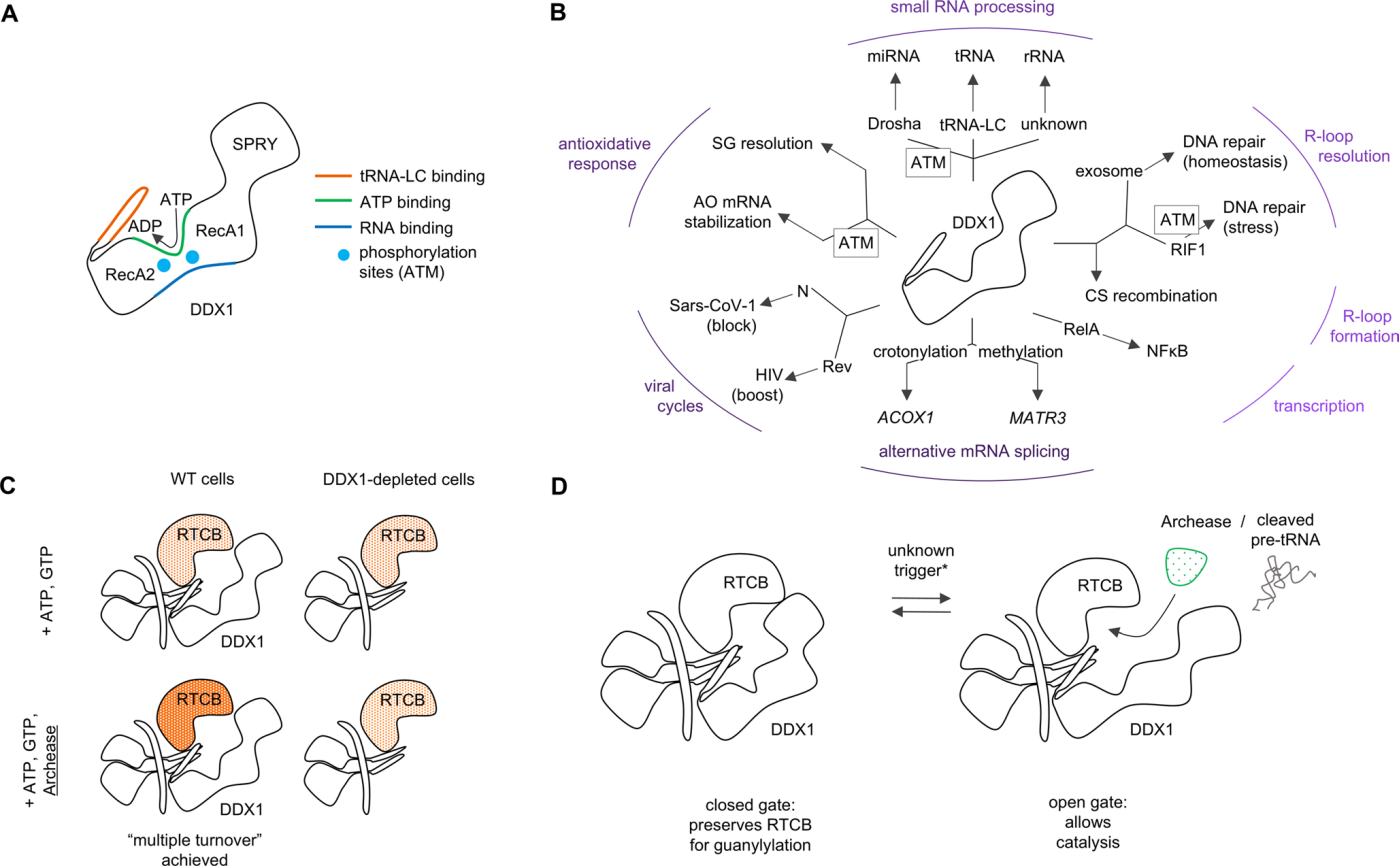
Overview of the enzymology of DDX1, its diverse cellular roles and its potential functions within the tRNA-LC. (**A**) Schematic representation of DDX1. DDX1 contains three domains: SPRY, RecA1 and RecA2; SPRY domain is inserted within the sequence of RecA1. The C-terminal helix (orange) is unique for metazoan DDX1 among the DEAD box proteins. Both ATP and RNA bind at the RecA1:RecA2 interfaces (green and blue, respectively), in a mutually cooperative manner. DDX1 can hydrolyse ATP and remodel nucleic acids, although its ability to unwind RNA helices is debated. Phosphorylation of DDX1 by ATM at one of the two identified sites (light blue) is critical for several of its functions, both in nucleus and cytoplasm. Note: sizes of the individual domains in the diagram are not necessarily in scale. (**B**) Best characterised functions of DDX1. SG: stress granules; AO: antioxidative; N: nucleocapsid protein; CS: class switch. These functions occur across cytoplasm and nucleus (see Fig. 4). (**C**) Extracts of wild-type cells and cells depleted of DDX1 display similar RNA ligase activity (top row); the addition of Archease to the extracts boosted the activity only in the wild-type extracts (bottom row). Intensity of the orange colour is proportional to the amount of the reaction product in the ligation assay. (**D**) DDX1, which is anchored to the tRNA-LC by the C-terminal helix (bundled with the helices of CGI-99 and a FAM98 paralogue), but otherwise appears flexible, may gate the RTCB active site, and only open it upon a yet unidentified trigger. Such gating may occur for example between dissociation of PYROXD1 and binding of Archease, and may be impaired in loss-of-function DDX1 constructs. While direct evidence is still lacking, this mechanism is proposed based on the experiments with recombinant tRNA-LC, where DDX1 appears dispensable for RTCB guanylylation, and the cryo-EM structure the piscine complex. * The unknown target could entail ATP binding or hydrolysis, RNA binding or a still elusive reaction

Conclusive experimental evidence support the activity of DDX1 as RNA-binding ATPase (Fig. 6A) [58, 148, 153, 154]. Single- and double stranded RNA, but also RNA-DNA hybrids and to a lower extent, double stranded DNA can all activate DDX1 [150]. Active sites for both ATP [58] and RNA [151] are at the interfaces between RecA1 and RecA2 domains of DDX1, located upstream of a C-terminal helix critical for the assembly of the tRNA-LC [110] (Fig. 6A). Binding of RNA to DDX1 strongly increases the affinity of DDX1 for ATP (and *vice versa*), thus boosting turnover at lower ATP concentrations; yet, such RNA-mediated boost does not substantially increase maximal velocity at saturating ATP conditions, which is moderate relative to homologous enzymes [154].

Interestingly, DDX1 displays exceptionally high affinity for reaction product ADP, which potentially “locks” it in the catalytically inactive state. To hypothetically achieve higher ATP to ADP turnover, as well as to facilitate ADP dissociation, DDX1 was thus proposed to be reliant on unknown cellular factors [154]. This may explain why many DDX1 functions lack biochemical reconstitution and hence, the mechanistic understanding of DDX1 functions remains poor. Indeed, despite the extensive knowledge of DDX1 biology, most phenotypes largely rely on DDX1 silencing and, occasionally, overexpression of catalytically dead Walker A motif constructs. Equally mysterious are the molecular bases for the pathogenicity of the microcephaly-associated variant V445I [155] and the potential role of DDX1 in neural crest pathologies [156].

### A complex member or a lone wolf? DDX1 between DNA repair and mRNA stabilisation

The first publications on DDX1 emerged in the early 1990s, with several reports observing its co-amplification with the known oncogene MYCN in primary neuroblastoma [157, 158], likely due to their chromosomal proximity [159]. The first specific published function entailed regulation of HIV Rev protein [102, 160]. DDX1 has since then been established to interact with Rev and to boost its nuclear RNA export activity, facilitating the HIV lifecycle [151, 161]. In contrast to its role towards HIV, DDX1 displays antiviral effect on SARS-CoV-2, where it binds nucleocapsid protein independently of RNA binding and ATP hydrolysis [128] (Fig. 6B, bottom left). DDX1 was also associated with transcriptional regulation [146] (Fig. 6B, bottom right) as well as rRNA processing [162] and biogenesis of specific microRNAs [163] (Fig. 6B, top). Looping back to the initial publications linking DDX1 to the oncogene MYCN, DDX1 has since emerged as a prognostic marker across multiple cancer types [164–167], and was proposed to promote the progression of testicular [168], colorectal [169], and non-small cell lung cancers [170] by regulating expression of genes critical for tumour stemness. Turning DDX1 into a cancer-specific pharmacological target may however be challenging due to its homology with other DEAD box proteins and its varied essential cellular functions.

With a particular focus on R-loop biology (Fig. 6B, right), DDX1 was peculiarly reported to facilitate both their formation during class switch recombination [147] and their removal during double stranded DNA break repair [124] [reviewed in [171]]. The role in DNA damage repair during genotoxic stress is facilitated by binding to RIF1 protein and by ATM-dependent phosphorylation [104, 172]. Most recently, it was proposed that DDX1 also plays a role in R-loop resolution under homeostatic conditions, through the interplay with exosomes [125].

DDX1 was also reported to partake in the formation of stress granules [112] in a manner independent of its RNA binding activity, with its silencing leading to delayed stress granule resolution [173]. The same report revealed that independently of stress granule localisation, DDX1 stabilises specific mRNA molecules during oxidative stress in cytoplasm (Fig. 6B, top left). Interestingly, and similarly to its nuclear roles in double stranded DNA break resolution, binding of DDX1 to such mRNAs critical for antioxidative response is also ATM dependent [174]. Failure of ATM to phosphorylate DDX1 triggers increase in oxidative stress, positioning DDX1 as a novel antioxidative regulator. The process has however not been reconstituted in vitro and how oxidative stress controls ATM-dependent DDX1 recruitment to mRNAs thus remains unknown.

Most recently, DDX1 was demonstrated to be regulated through EZH2-mediated lysine methylation [175], turning it into second subunit of the expanded tRNA-LC to be affected by methylation mechanisms alongside FAM98A [129]. Unlike oxidative stress, DDX1 methylation disrupts mRNA binding. Dysregulation of DDX1 methylation is proposed to contribute to intervertebral disc degeneration through aberrant alternative splicing of *MATR3* gene [175] (Fig. 6B, bottom). Finally, a different post-translational modification, crotonylation, enables DDX1 to sustain peroxisomal function through *ACOX1* alternative splicing [176].

Whether DDX1 performs the described roles on its own was cast in doubt when the protein was identified as a core subunit of the tRNA-LC, apparently unstable in the absence of the other complex members [52, 110] (see Sect. 3). The roles of the other subunits in the above listed processes still need to be tested.

### (Not) a guanylylation factor? The role of DDX1 within the tRNA-LC

Within the tRNA-LC, DDX1 was originally described as catalytically non-essential [52], followed by the observation that it is critical for the tRNA-LC activity under “multiple turnover conditions” [58]. This referred to the conditions where the tRNA-LC is able to perform the entire catalytic cycle (see Fig. 1F) in the assayed conditions, rather than only “finishing” the cycles that had been initiated by guanylylation of RTCB before cell lysis. Namely, the extracts of cells silenced for DDX1 displayed lower RNA ligation activity than the wild-type extracts only when Archease was added to the reaction (Fig. 6C). Similarly, tRNA-LC immunoprecipitated from cells overexpressing catalytically inactive DDX1 displayed lower ligation activity than a complex with wild-type DDX1 when Archease was added. This led to the initial characterisation of DDX1 as a partner of Archease, i.e. a protein directly involved in RTCB guanylylation [58].

However, new evidence, including enzymatic assays performed with recombinant RTCB and the tRNA-LC purified from insect cells [74, 75] challenge a direct and universal role of DDX1 in guanylylation of RTCB. Recombinant RTCB can be efficiently guanylylated in vitro in the absence of any other expanded subunit of the tRNA-LC except Archease. In fact, in a separate study RTCB displayed higher activity in the absence of DDX1, suggesting a potential inhibitory role of DDX1 [72]. The authors suggest that this inhibition occurs due to DDX1 partially overlapping with Archease and/or with the RNA binding site in RTCB, as suggested by the density observed upon cryo-EM analysis of the piscine complex (Fig. 6D). Finally, inducible genetic depletion of DDX1 seemed not to affect *Xbp1* mRNA splicing, contrary to the expectation in case DDX1 played a critical role in the guanylylation cycle [152].

It is of note that oxidation of DDX1 was observed alongside RTCB, albeit at slightly higher oxidant concentrations [31]. Furthermore, the full tRNA-LC appeared more resilient to oxidative inactivation when compared to RTCB alone, suggesting a potential unknown redox-related role of DDX1 in the tRNA-LC biology. Finally, a function more in line with the nucleic acid chaperone and putative helicase nature of DDX1, where it acts in substrate remodelling, cannot be ruled out.

These findings imply a likely more indirect role of DDX1, where rather than being catalytically involved in RTCB guanylylation, it may sustain the tRNA-LC in the guanylylation-ready state over time or “chaperone” (re)folding of selective RNA substrates. It remains to be uncovered (1) how this occurs, (2) whether mechanisms that alleviate its potential inhibitory effect and steric clashing with Archease and RNA exist, and (3) ultimately, whether these mechanisms affect other reported functions of DDX1.

## The end of the cycle: protection, inactivation, and degradation of the tRNA-LC

No feature of the tRNA-LC more evidently demonstrates the struggle to sustain an ancient, metal ion-based active site than the propensity of RTCB for oxidative inactivation. Such sensitivity triggered a sprawling network of safeguarding mechanisms in modern aerobic *Eukarya*, involving prevention, repair and potentially degradation of the damaged tRNA-LC.

**Table:**
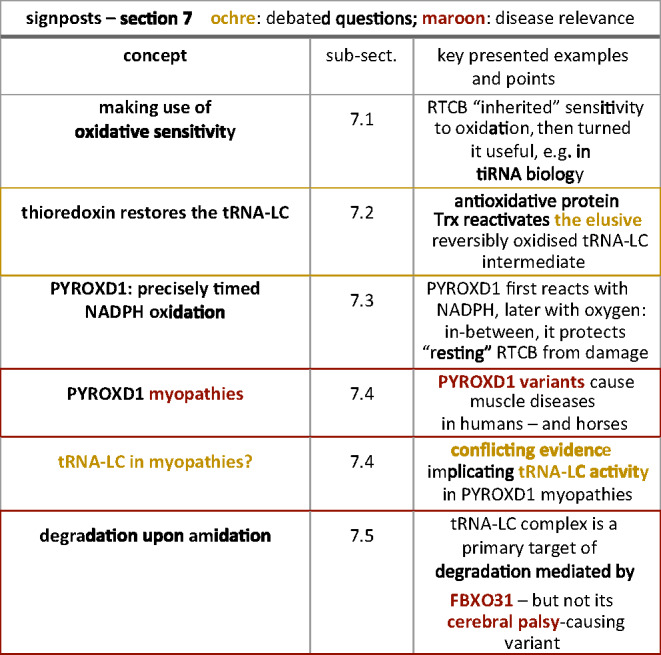

### Oxidative inactivation: evolutionary relic or regulatory mechanism?

**Fig. 7:**
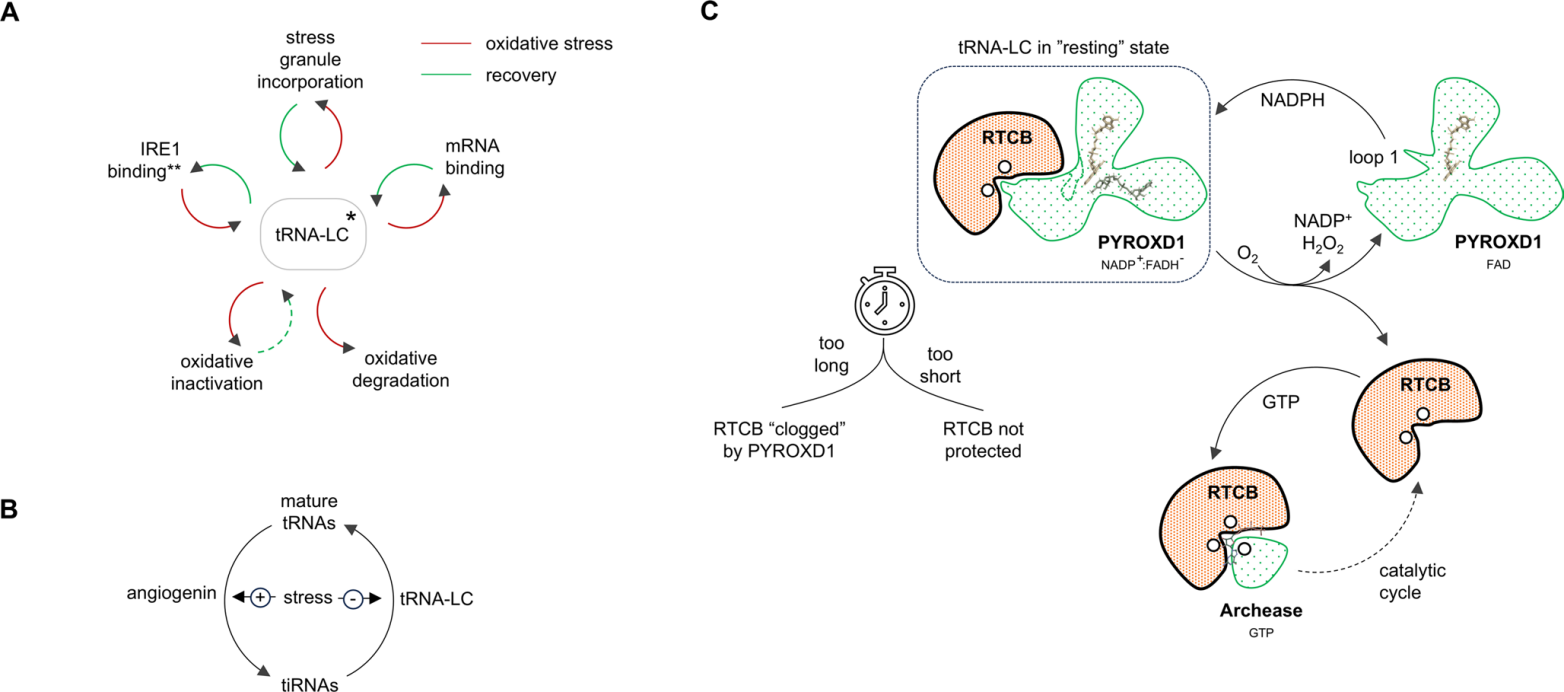
Redox-regulated properties of the tRNA-LC, potential benefits of the oxidative inactivation of the tRNA-LC in the biology of tiRNAs, and its protection by PYROXD1. (**A**) Redox-regulation has been implicated in at least five aspects of the biology of the tRNA-LC. * Individual phenotypes were only associated with a subset of the subunits of the expanded tRNA-LC, while the potential involvement of the other subunits often remains unexplored. ** Regulation through redox-mechanisms of the RTCB-IRE1 interaction has not yet been directly demonstrated; however, it was shown to be controlled through (de)phosphorylation of RTCB by PTP1B and c-Abl, canonical targets of redox-regulation. DDX1 binds and stabilises mRNAs involved in antioxidative response. Multiple subunits, in particular of the FAM98A-containing complex, were reported in stress granules. Oxidative inactivation presumably targets the cysteine residue in the active site of RTCB, and finally, degradation is initiated by C-terminal amidation of a currently unknown subunit. Red and green arrows point towards the processes that occur during oxidative stress and recovery, respectively. It is unknown how different regulatory events are synchronised. (**B**) The tRNA-LC is proposed to seal the ends of angiogenin-generated tiRNAs, an undesired reaction that can be prevented during stress through its oxidative inactivation. Importantly, angiogenin activation and tRNA-LC inactivation are triggered proteotoxic and oxidative stress, respectively; such stresses would thus need to co-occur for the depicted cooperation to take place. (**C**) The oxidoreductase PYROXD1 in the NADP^+^:FADH^−^-bound conformation is part of the expanded tRNA-LC in its “resting state” (top left). Loop 1 becomes disordered upon formation of NADP^+^:FADH^−^ complex within PYROXD1 (shown in dotted lines and “inside” the protein), removing steric hindrance and allowing binding of RTCB. In the FAD state (top right), loop 1 forms a structured pin on the *si*-side of the FAD ring, thus previously described as *si*-pin. The NADP^+^:FADH^−^ complex undergoes oxidation to allow PYROXD1 dissociating from RTCB and Archease to bind in its place, initiating the catalytic cycle (bottom). The lifetime of the tRNA-LC in the resting state is strictly regulated, as its prolongation would disable Archease binding, and its shortening would make the tRNA-LC vulnerable to oxidation (left). While the scheme displays PYROXD1:RTCB complex, this interaction occurs within the context of the wider expanded tRNA-LC [31, 111]; the remaining subunits, which presumably do not directly interact with PYROXD1, are not shown for simplicity

We have so far described three mechanisms that regulate the tRNA-LC under oxidative stress: migration of the FAM98A-containing complex to the stress granules [112], binding of DDX1 (potentially within the tRNA-LC) to specific mRNAs encoding for antioxidative proteins [173, 174] and presumably, dissociation of the tRNA-LC from IRE1 mediated by RTCB (de)phosphorylation [136]. Two mechanisms (see below) however lead to even more drastic effects: abolishment of its enzymatic activity and its proteolytic degradation (Fig. 7A).

Reactive oxygen species in the presence of trace copper ions lead to a potentially total loss of the tRNA-LC activity [31]. This reaction presumably occurs via oxidation of the active site cysteine and the concomitant loss of catalytic metal ions, as evidenced by the metal-free, oxidised preparation of *Pyrococcus horikoshii* RtcB [76] (see Sect. 5). Notably, this is an obligatory anaerobic organism, and hence such inactivation is unlikely to play a role in vivo in its native environments.

In contrast, sensitivity to oxidative inactivation is a central pillar of the biology of the tRNA-LC in animals. Loss of activity mediated by oxidative stress is evident both in cell extracts, as well as through diminished UPR response and the accumulation of tRNA fragments in cells and in a mouse model [31]. Oxidation of the tRNA-LC occurs under basic cell culture conditions without the bulk addition of oxidative stressors, witnessing to high oxidative sensitivity of the catalytic system. This is most obviously observed through addition of the antioxidant N-acetyl cysteine (NAC), which boosts the activity of the tRNA-LC under otherwise standard cell culture conditions [31]. Newest findings revealed that not only inactivation, but also degradation of the tRNA-LC is likely triggered by metal-ion based oxidation events [177]. Whether inactivation and degradation of the tRNA-LC are coupled remains unknown.

The inherent fragility of the tRNA-LC towards oxidation may however not only be a liability. Instead, it was proposed that human cells utilise this sensitivity to regulate the amounts of angiogenin-induced tRNA fragment [30]. Under varying stress conditions, the endonuclease angiogenin cleaves mature tRNAs [178], generating a distinct set of cytoprotective tRNA fragments known as tRNA-derived stress/angiogenin-induced RNAs (tiRNAs) [179, 180]. Re-ligation of tiRNAs by the tRNA-LC would thus be counter-productive, and indeed, silencing of RTCB results in increased amounts of fragments [30]. It has thus been proposed that oxidative inactivation mimics the silencing effect, providing tiRNAs with a two-tiered boost upon stress: generation by angiogenin, and preservation through RTCB oxidative inactivation (Fig. 7B). It is important to emphasise that the stress-inducing agents used to selectively activate angiogenin (e.g. sodium arsenite) and to inactivate the tRNA-LC (e.g. hydrogen peroxide) differ in mechanism of action, implying that the cooperativity between the two processes may only arise under mixed-stress conditions when both proteotoxic and oxidative stress occur simultaneously [30, 181].

More generally, while the oxidative inactivation of the tRNA-LC has been dissected in detail, the same is not true for the physiological potential of such regulation. Do changing oxidative stress levels in, for example, ovulation, exercise, immune response, or ultimately ageing, affect tRNA-LC and concomitantly the levels of tRNA-derived fragments, and how they interplay with such processes remains a fully unexplored avenue.

**Table:** 

Oxidative boost?	
Surprisingly, both in the context of the UPR and in vitro, a narrow window of small amounts of oxidants was observed to slightly boost the ligation activity of the tRNA-LC [10, 87]. What is the mechanism of such boost and whether it is physiologically relevant is still to be explored. This article however focuses on the more pronounced and robust effect of the oxidation-induced inactivation of the tRNA-LC and the mechanisms for its prevention or repair.	

So far, three mechanisms have been described to mitigate the sensitivity to oxidation and thus sustain the activity of the tRNA-LC under homeostatic conditions. RTCB guanylylation, described above, leads to an increased resistance to oxidative inactivation [120]. The mechanism of this effect is not yet elucidated, and may be linked to steric hindrance of the metal ion active site, or to the lowered occupancy of the presumably more dynamic metal site B, which lowers the risk of copper binding (see Sect. 5).

### To the oxidised state and back: thioredoxin comes to rescue

The second mechanism deals with repair, rather than the prevention of oxidative damage. Silencing of thioredoxin (Trx), a prominent small antioxidative protein in animals, increases the sensitivity of the tRNA-LC to oxidation and hinders its reactivation upon cease of stress [32]. This occurs only with concentrations of oxidants that trigger partial loss of the tRNA-LC activity; total loss appears irreversible, in line with overoxidation of the active site cysteine (see Fig. 5B). A thioredoxin construct engineered with a cysteine replacement so that it traps, rather than fully reduces, its targets, captures the tRNA-LC upon oxidative stress. These data imply that thioredoxin can directly reduce the reversibly oxidised tRNA-LC.

It is important to note that the exact identity of this partially oxidised species within the tRNA-LC is as yet unknown. This is particularly interesting having in mind that metal ions often trigger formation of more “reactive” oxidants [182–184]. Such species can cause severe damage, including thiyl-radical mediated cysteine overoxidation and even protein degradation [185, 186].

Trx can thus revert partial and yet largely mechanistically unexplored damage to the tRNA-LC, while guanylylation protects it during the catalytic cycles. One protein however evolved with the purpose of stemming oxidative inactivation in the first place: PYROXD1.

### PYROXD1 and the “resting” tRNA-LC: the Art of perfect timing

Approximately one in twenty proteins essential for the survival of animal cells remain without any functional annotation [187]. Until recently, that was the case of PYROXD1, the most recently described subunit of the expanded tRNA-LC.

PYROXD1 is homologous to the textbook enzymes such as ferredoxin reductases and thioredoxin reductases [31]. Just like these enzymes, PYROXD1 contains two small molecule binding sites: prosthetic group FAD-binding site, and a site for the reversibly bound cofactor NADPH. Of note, PYROXD1 can use both unphosphorylated (NADH) and phosphorylated (NADPH) forms of the dinucleotide. NADPH, the dominant reduced nicotinamide adenine dinucleotide outside of mitochondria [188, 189], is however the likely cofactor due to the nucleo-cytosolic localisation of PYROXD1 and RTCB [139].

PYROXD1 catalyses the oxidation of NAD(P)H to NAD(P)^+^, with concomitant reduction of FAD to FADH^−^. This reaction entails a hydride (H^−^) transfer, and leads to the loss of distinct yellow colour of the recombinant PYROXD1 originating from oxidised FAD. The process results in the formation of NADP^+^:FADH^−^ charge transfer complex (CTC) [31] (Fig. 7C, top).

The next step is where PYROXD1 diverges from many of its homologues. Unlike most disulphide reductases and related flavoenzymes [190], PYROXD1 does not have a cysteine-containing loop covering the active site, rendering it inactive for reduction of disulphides [31, 120]. This is in contrast with its annotated name, “pyridine nucleotide-disulphide oxidoreductase domain-containing protein 1” (Uniprot). The NADP^+^:FADH^−^ complex within PYROXD1 can be oxidised by O_2_, so far the only physiological substrate of PYROXD1. Here, H^−^ is transferred from FADH^−^ to O_2_ to ultimately generate H_2_O_2_. In the process, FAD is regenerated and NADP^+^ dissociates as a reaction product [31] (Fig. 7C, top). However, the flavin group of PYROXD1 is shielded from the access of potential electron acceptors or other reactants. This is achieved by an active site tryptophane residue prior to the reaction with NADPH [31], and by a partially disordered loop after the formation of the NADP^+^:FADH^−^ complex [120]. This steric shielding renders oxidation of the CTC very slow, providing unusually long life to the form of PYROXD1 containing a NADP^+^:FADH^−^ complex.

Such “slowness” is key for the protection of RTCB. Formation of the CTC triggers a conformational change in PYROXD1 [191]. This change entails that the pin hovering above the si-side of the flavin ring and sterically preventing binding of RTCB [“si-pin” [31], subsequently annotated as loop 1] assumes a disordered conformation (Fig. 7C, bottom). Once this steric hinderance is overcome, PYROXD1 binds RTCB. Critically, PYROXD1 inserts the C-terminal tail deep into the active site of RTCB, ultimately reaching the metal ions and blocking access to reactive oxygen species. This binding cannot however be unlimited, as it would sterically prevent binding of Archease [75] and RNA [76]; hence, the NADP+:FADH- complex ultimately oxidises, PYROXD1 returns to NADPH-free conformation, and dissociates (Fig. 7C, bottom). This protects RTCB in its resting state, while giving it windows of opportunity between PYROXD1 dissociation and re-association to undergo guanylylation and perform its catalytic cycle. This balance is key to providing sufficient protection to the tRNA-LC without blocking its activity, allowing sustenance of pre-tRNA splicing and UPR, both of which are crippled upon PYROXD1 silencing in human cells [31].

**Table:** 

Antioxidative NADP+?	
Before the structures of the RTCB:PYROXD1 complex [112] and the “resting” tRNA-LC ([136] emerged, a complementary, NAD(P)^+^-based protective mechanism was proposed [10]. This model was primarily based on the observations that: 1) PYROXD1 binds the tRNA-LC in the presence of NAD(P)H, 2) PYROXD1 oxidises NAD(P)H to NAD(P)^+^ in a manner impaired in some disease-causing variants, and 3) critically, NAD(P)^+^ desensitises the tRNA-LC towards oxidative inactivation while NAD(P)H makes it more susceptible. The latter effect in particular was attributed to the interplay between NAD(P)H, metal ions and Fenton-like reactions [145]. Recent structural and reconstitution data (see above) suggest that this is not the primary mechanism by which PYROXD1 protects the tRNA-LC. Whether and how PYROXD1 affects the proximal NAD(P)H levels in vivo, and to which extent the cellular or local increase in NAD(P)^+^/NAD(P)H ratio reduces the sensitivity of the tRNA-LC to oxidative damage (for example during starvation) remains to be determined. Finally, whether this interplays with the alternative, NAD^+^-dependent “yeast-like” RNA ligation pathway, particularly in muscles (see section 2), is also to be dissected.	

### An emerging oxidoreductase: PYROXD1 in disease

PYROXD1 has recently been emerging as the latest protein in the expanded tRNA-LC with relevance in the context of human diseases. Changes in PYROXD1 expression were observed to correlate with a number of psychiatric conditions, including panic disorder and major depressive disorder [193, 194], as well as in colorectal cancer [195]; metastatic ability of laryngeal squamous cell carcinoma was shown to be modulable through PYROXD1 expression, presumably via interaction with an established oncogene, ROCK [196].

However, the largest body of causative evidence links PYROXD1 with genetic muscle disorders. Multiple variants of PYROXD1 cause muscle diseases in humans [139–141, 197–200] and horses [201], including congenital myopathy and limb girdle muscle dystrophy. Affected probands are either homozygous or compound heterozygous for missense variants (six identified to date), or they carry a missense and a loss-of-function (frameshift or exon-skipping splice) variant. Most recently, patients with combined muscle and connective tissue presentation were also observed to harbour two PYROXD1 variants [202]. Despite mounting evidence, no dedicated review on muscle disorders and wider pathobiochemistry caused by PYROXD1 variants has emerged so far.

The molecular basis of these disorders remains largely unknown. Mitochondria appear affected in cells with disruptions in PYROXD1 [139–141]. While PYROXD1 can complement glutathione-reductase in antioxidative defence when ectopically expressed in yeast, multiple disease-causing variants (N155S, Q372H, Y354C) partially fail to do so. The link to mitochondria, as well as the basis of putative antioxidative activity of PYROXD1, remain puzzling.

Two initially reported pathogenic PYROXD1 variants (N155S, Q372H) display defective protection of the tRNA-LC upon in vitro reconstitution [31]. The reconstituted effects of the more recently reported variants on RNA ligation activity have not yet been reported. However, recent findings challenge the idea that all pathogenic PYROXD1 variants fail to bind the tRNA-LC and therefore sustain all its functions. The newest reported variant ΔE496 appears to form a complex with RTCB *in vitro* [120]. Furthermore, the UPR appears unaltered in cells generated from multiple unrelated PYROXD1-linked patients [202].

*Xbp1* mRNA splicing is however very robust, and even remains unaffected upon silencing of RTCB [99], potentially due to compensation from a putative “yeast-like” system (see Sect. 2.2). The faults in RNA splicing in patients may thus only be revealed in pre-tRNA splicing rather than *Xbp1* mRNA splicing, in specific cell types or under conditions of stress.

It therefore remains unclear whether the disruption of non-canonical RNA splicing or of another, as of yet unknown role of PYROXD1, lies at the core of PYROXD1-related pathologies. Its closest paralogues in humans are the apoptosis inducing factors (AIFs), which have been studied for decades before their roles in assembly and regulation of respiratory chain complexes [191, 203, 204] and suppression of ferroptosis [205, 206] were revealed. Further studies are thus required to address whether pre-tRNA splicing displays a phenotypic defect in all probands with PYROXD1 variants, if PYROXD1 has additional roles, and how deregulations of both the known and the potential unknown functions could result in disease phenotypes.

### Death of the tRNA-LC: a novel protein degradation mechanism

The newest twist in the enzymology of the tRNA-LC arose from an unexpected angle. It has emerged that proteins that end with C-terminal amides, rather than the canonical carboxylates, effectively differing in two atoms in the entire polypeptide sequence, are recognised and degraded in the cell [177]. The primary target of this pathway is, surprisingly, the tRNA-LC in its FAM98A-containing form.

It is unknown how an amide emerges in one of the subunits of the tRNA-LC. Such chemistry can result from Fenton-like reactions, whereby highly “reactive” species accumulate in the presence of reduced redox-reactive metals and hydrogen-peroxide [182, 183]. Indeed, in vitro reconstitution of the Cu-mediated reaction in the presence of the tRNA-LC resulted in the accumulation of C-terminal amides in several subunits [177]. It is however unclear if this is the mechanism that triggers degradation of the tRNA-LC in cells, and critically, why is the tRNA-LC so prominently targeted. While a similar Fenton-like chemistry may occur in the RTCB active site during irreversible oxidative inactivation, it remains unknown whether these processes are mechanistically coupled. Furthermore, it is unclear whether the recently identified deubiquitylation of RTCB and DDX1 by USP45 [207] prevents proteasomal degradation of the tRNA-LC flagged through such amide-mediated ubiquitylation, or rather acts on another tRNA-LC pool. Further exploration of both oxidative inactivation and the presumably oxidative degradation will be necessary to understand both processes and their implications.

## Open questions and outlook

Recent years have brought a rapidly increasing understanding of the tRNA-LC and the functions of its subunits. Yet, they have also highlighted the gaps and contradictions on several key points, and paved the way for the so far poorly addressed translational approaches. A **systematic issue** in the field is whether (and if so, which) other components of the tRNA-LC contribute to the numerous functions so far attributed to individual subunits. From an **evolutionary perspective**, a comprehensive analysis of phylogenetic distribution of the tRNA-LC subunits is required, with special emphasis on the species where different subunits do not co-appear. Links between the subunits of the tRNA-LC and mitochondria throughout evolution are also particularly puzzling. **Physiological roles** of both inactivation and clearance of the oxidatively damaged tRNA-LC remain largely unstudied. On the **structural and mechanistic aspects**, detailed information on binding interface with RNA substrates is lacking; the enzymatic activity(ies) of DDX1 and its role within the tRNA-LC are being re-addressed; the identity of the reversibly oxidised tRNA-LC state is mysterious, as is the complete picture regarding how many and which metal ions bind the complex simultaneously during “resting” and catalysis. The **molecular basis of the diseases** linked to the tRNA-LC and the functionally adjacent proteins remains a largely unexplored avenue: why do PYROXD1 variants lead to myopathies, CLP1 variants to neurodegeneration, and DDX1 variants (potentially) to microcephaly? Is there a causation between FAM98B sequestration and NIID or FXTAS? Do these diseases share hallmarks and mechanisms, and thus potential therapeutic approaches? As structural and biochemical information is steadily increasing, such translational questions are growing in urgency and relevance in the research on the tRNA-LC.

## Data Availability

As a review article, this manuscript does not include any previously unpublished data.
